# Machine Learning Exploration of Food-Derived Chemical Space for Potential Nutritional Metabolic Regulators Targeting Dipeptidyl Peptidase-4

**DOI:** 10.3390/ph19030349

**Published:** 2026-02-24

**Authors:** Nada A. Alzunaidy

**Affiliations:** Department of Food Science and Human Nutrition, College of Agriculture and Food, Qassim University, Buraydah 51452, Saudi Arabia; n.alznedy@qu.edu.sa

**Keywords:** food-derived compounds, machine learning identification, dipeptidyl peptidase-4 (DPP4), dietary supplements, metabolic regulation

## Abstract

**Background:** Dipeptidyl peptidase-4 (DPP4) is a key metabolic enzyme involved in postprandial glucose regulation through incretin hormone modulation, making it an important target in nutrition and metabolic health research. Although dietary and plant-derived bioactive compounds have been reported to influence DPP4, exploration of the food-associated chemical space remains limited by its size and diversity. **Methods:** Here, we present an integrated computational framework combining machine learning, molecular docking, and molecular dynamics simulations to prioritize dietary and supplemental compounds with potential interaction capacity toward DPP4. Supervised classification models were trained on a curated DPP4 bioactivity dataset and evaluated using scaffold-based partitioning to ensure chemically realistic generalization. **Results:** The top-performing random forest model achieved robust performance across independent splits (mean AUC 0.889 ± 0.017; average precision 0.959 ± 0.010) and was applied to screen 69,574 food-derived compounds. Model interpretation identified recurring heteroaromatic and polar substructural features associated with predicted interaction propensity. Structure-based screening further prioritized seven food-derived compounds, including lipid-associated coenzyme A derivatives, which occupied the canonical DPP4 binding site with favorable docking scores (−13.12 to −12.06 kcal/mol). Extended molecular dynamics simulations (500 ns) demonstrated stable binding geometries, compact hydrogen-bond networks, and consistent engagement of key DPP4 residues, including Glu205, Glu206, Arg125, and Tyr631. **Conclusions:** Overall, our study provides a scalable computational strategy for identifying bioactive dietary and supplemental compounds with potential relevance to metabolic regulation. The framework supports nutraceutical research and functional food development by enabling targeted experimental investigation of diet–enzyme interactions.

## 1. Introduction

Diet-associated metabolic dysregulation has become increasingly prevalent across populations, with exaggerated postprandial glucose excursions emerging as a consistent feature of contemporary dietary patterns [1]. Conventional nutritional frameworks have largely emphasized macronutrient composition and energy balance, yet these approaches only partially account for the marked interindividual variability observed in post-meal metabolic responses [2]. Accumulating evidence indicates that meals deliver a complex mixture of low-molecular-weight bioactive constituents capable of influencing metabolic processes beyond the effects of carbohydrates, fats, and proteins alone [3]. In this context, postprandial metabolism is increasingly understood as a dynamic, enzyme-mediated system in which dietary chemistry modulates hormonal signaling, substrate handling, and glucose homeostasis over short temporal scales [4]. Such a perspective positions metabolic regulation as an emergent property of interactions between dietary compounds and endogenous enzymatic networks rather than a passive consequence of nutrient load alone [5].

At the molecular level, dietary exposure entails continuous contact between food-derived small molecules and endogenous enzymes that govern digestion, nutrient absorption, and postprandial hormone turnover [6]. These enzymes operate as nutrient-response regulators that integrate chemical cues from the diet into coordinated metabolic signaling, rather than as fixed pharmacological targets [7]. Interactions involving food-derived compounds are typically characterized by low affinity, reversibility, and strong dependence on concentration, timing, and dietary context, distinguishing them from the sustained binding profiles associated with therapeutic agents [8]. Within this framework, food chemistry contributes to metabolic regulation by subtly shaping enzymatic activity and signaling fluxes during the fed state, rather than through direct enzyme blockade [9]. An enzyme-centered perspective therefore provides a mechanistically grounded approach for studying nutritional modulation while remaining aligned with the transient and context-dependent nature of dietary exposures [10]. Within this nutritional framework, dipeptidyl peptidase-4 (DPP4) occupies a central position in the regulation of postprandial endocrine responses [11]. DPP4 is widely expressed and rapidly engages following food intake, where it governs the turnover of incretin hormones that coordinate insulin secretion, glucagon suppression, and glucose disposal during the fed state [12]. Rather than representing a pathological node, DPP4 functions as a metabolic checkpoint that fine-tunes hormonal signaling in response to meal composition and timing [13]. Consistent with this role, a growing body of nutritional and biochemical evidence indicates that dietary peptides, flavonoids, and polyphenolic compounds can influence DPP4-associated pathways under physiological conditions, often in a manner dependent on food matrix, co-ingestion, and digestive processing [14]. DPP4 was selected as a nutritionally relevant enzymatic target due to its central role in postprandial hormone regulation and glucose homeostasis [15]. Framed in this way, DPP4 provides a mechanistically coherent link between dietary chemical diversity and short-term metabolic regulation without invoking disease-centered or therapeutic interpretations [16].

The chemical landscape of the human diet encompasses thousands of structurally diverse small molecules originating from plant, animal, and microbial sources, many of which remain incompletely characterized at the molecular level [17]. Systematic experimental evaluation of this breadth of dietary chemical space is constrained by practical limitations, including compound availability, matrix complexity, and the transient nature of postprandial exposure [18]. In this situation, computational approaches offer a complementary strategy for organizing and interrogating large collections of food-derived compounds [19]. Machine-learning methods, when applied with appropriate restraint, enable the identification of recurring structural features and interaction patterns associated with enzymatic engagement, thereby supporting hypothesis generation in nutritional biochemistry [20]. Importantly, such models are not intended to predict efficacy, potency, or physiological outcomes, but rather to prioritize and contextualize dietary compounds based on shared chemical characteristics relevant to enzyme interaction propensity [21]. This approach provides a scalable means of exploring nutritional chemical diversity while maintaining clear boundaries between predictive modeling and experimental validation [22].

**Figure 1:**
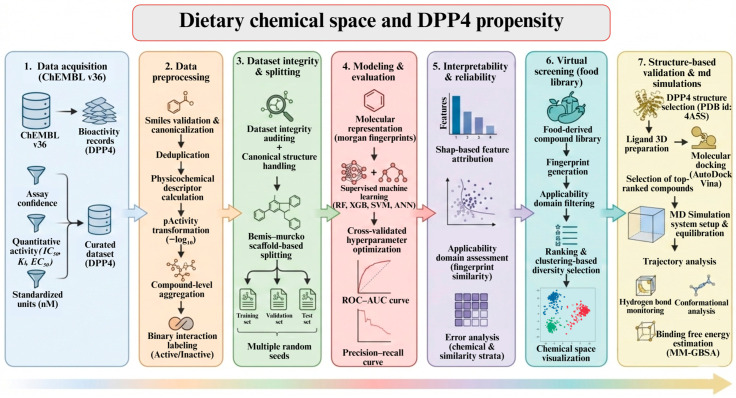
Workflow for dietary chemical space mapping and DPP4 interaction propensity. Bioactivity data for DPP4 were curated from ChEMBL v36 and standardized prior to molecular descriptor generation. Scaffold-aware dataset splitting enabled supervised machine-learning model development and evaluation, followed by interpretability and applicability-domain assessment. Food-derived compounds were subsequently screened in silico, and top-ranked candidates were subjected to structure-based validation and molecular dynamics simulations to assess binding stability and energetics.

Accordingly, the present study is framed to examine dietary chemical space through the lens of molecular interaction propensity rather than enzymatic inhibition and therapeutic modulation [23]. This study examines food-derived chemical structures as part of everyday dietary exposure. Using machine-learning-based structure–activity modeling together with curated biochemical data, we explore how recurring structural features in dietary compounds may relate to interactions with dipeptidyl peptidase-4 under physiological conditions. The analysis is intended to organize and interpret dietary chemical space from a mechanistic perspective while recognizing that in silico predictions are hypothesis-generating rather than confirmatory. Within this framework, the objective was to identify food-derived compounds whose structural characteristics were associated with a higher likelihood of interacting with DPP4, providing a basis for targeted experimental investigation in a nutritional context. Bioactivity data targeting dipeptidyl peptidase-4 (DPP4) were obtained from ChEMBL v36 [24], curated, and standardized prior to molecular representation using circular fingerprints (Figure 1). To promote chemically realistic generalization, scaffold-based dataset splitting was implemented during supervised model development. Model interpretability and reliability were evaluated using feature attribution analysis and applicability-domain assessment. The validated models were then applied to a large food-derived compound library for virtual screening, followed by structure-based docking and molecular dynamics simulations to characterize the binding stability and interaction energetics of prioritized candidates. Molecular representation for supervised learning was based on circular fingerprints together with standard physicochemical descriptors, enabling the integration of substructural patterns and global molecular properties within the classification framework.

## 2. Results

### 2.1. Assembly and Physicochemical Characterization of the DPP4 Bioactivity Dataset

**Figure 2:**
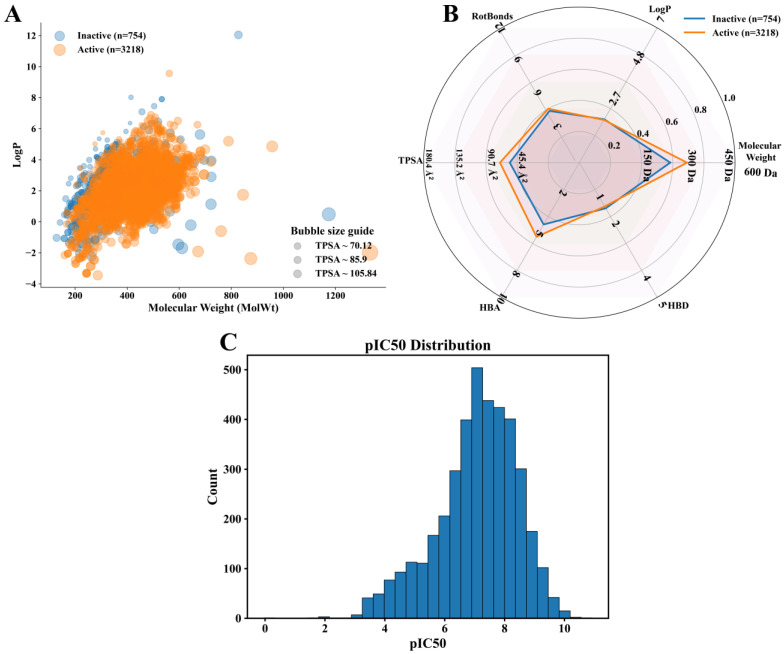
Physicochemical landscape and activity distribution of the curated DPP4 dataset. (**A**) Distribution of interaction-positive (*n* = 3218) and interaction-negative (*n* = 754) compounds plotted as molecular weight versus calculated LogP. Bubble size represents the topological polar surface area, illustrating the spread of polarity across chemical space. (**B**) Radar plot comparing normalized median physicochemical properties of interaction-positive and interaction-negative compounds, including molecular weight, LogP, hydrogen bond donors, hydrogen bond acceptors, topological polar surface area, and number of rotatable bonds. Values were scaled to enable a direct comparison across descriptors. (**C**) Distribution of pIC_50_ values for interaction-positive compounds following activity standardization and aggregation at the compound level, showing a unimodal activity profile across the dataset.

A curated reference dataset describing chemical interaction profiles with dipeptidyl peptidase-4 (DPP4) was assembled from ChEMBL using the target identifier CHEMBL284. Following filtering and standardization procedures described in the Methods, the final compound-level dataset comprised 3972 unique molecules, of which 3218 were classified as interaction-positive and 754 as interaction-negative based on the predefined pIC_50_ threshold. All compounds were associated with validated concentration-based bioactivity measurements and had computable molecular descriptors. To contextualize the chemical space represented in the curated dataset, global distributions of molecular weight, lipophilicity, and polarity were examined. A bivariate projection of molecular weight against calculated LogP revealed substantial overlap between interaction-positive and interaction-negative compounds across the dominant region of chemical space (Figure 2A). Both classes were primarily distributed between approximately 200 and 600 Da, with LogP values spanning from low to moderately lipophilic ranges. No abrupt separation between classes was observed along either axis, indicating that interaction status could not be attributed to a single physicochemical dimension. Bubble scaling by topological polar surface area further indicated broad TPSA variability within both classes, without discrete clustering. Aggregate physicochemical profiles were compared using normalized radar representations (Figure 2B). Interaction-positive compounds exhibited modest shifts toward higher median molecular weight, lipophilicity, hydrogen bond acceptor count, and topological polar surface area relative to interaction-negative compounds. In contrast, hydrogen bond donor counts and rotatable bond numbers showed largely overlapping distributions between the two groups. These differences were gradual rather than categorical, reinforcing that the curated dataset reflects continuous variation in physicochemical properties rather than distinct drug-like versus non-drug-like regimes. The distribution of standardized activity values for interaction-positive compounds was examined following transformation to pIC_50_ (Figure 2C). The resulting distribution was unimodal, centered in the mid-to-high micromolar to low nanomolar range, with a gradual decline toward higher potency values. No secondary modes or discontinuities were observed, consistent with aggregation across heterogeneous assay formats and chemical series. The smooth distribution supported the use of a single interaction threshold for downstream classification without introducing artificial stratification. These analyses confirm that the curated CHEMBL284 dataset spans a chemically diverse yet coherent interaction space, with substantial overlap in global physicochemical properties between interaction-positive and interaction-negative compounds. This structure is consistent with a nutritionally and chemically heterogeneous reference set and provides an appropriate foundation for subsequent structure–activity modeling.

### 2.2. Scaffold-Based Partitioning and Dataset Composition Across Independent Split Seeds

To evaluate model performance under conditions of reduced structural overlap, the curated DPP4 dataset was partitioned using a scaffold-based splitting strategy. Compounds were grouped by Bemis–Murcko scaffolds derived from canonicalized structures, and all molecules sharing the same scaffold were assigned to the same subset. This procedure ensured that closely related chemical series were not distributed across training and evaluation partitions. To assess the stability of downstream analyses with respect to stochastic scaffold assignment, the splitting procedure was repeated using three independent random seeds. Each seed generated a complete and non-overlapping partition of the dataset into training, validation, and test subsets while preserving scaffold integrity. Across all three seeds, the overall distribution of compounds and scaffolds across subsets remained consistent, indicating that the partitioning strategy was robust to seed choice and did not introduce systematic bias. Seed-specific statistics describing the number of compounds, scaffold counts, and class composition within each subset are reported in Supplementary Tables S1–S3, corresponding to seeds 1, 2, and 3, respectively. These tables document the reproducibility of the scaffold-based splitting process and provide full transparency regarding dataset composition used for model training and evaluation. All subsequent modeling results were evaluated independently across the three scaffold split seeds and summarized across seeds to account for partition-level variability.

### 2.3. Supervised Classification Performance Under Scaffold-Based Evaluation

**Figure 3:**
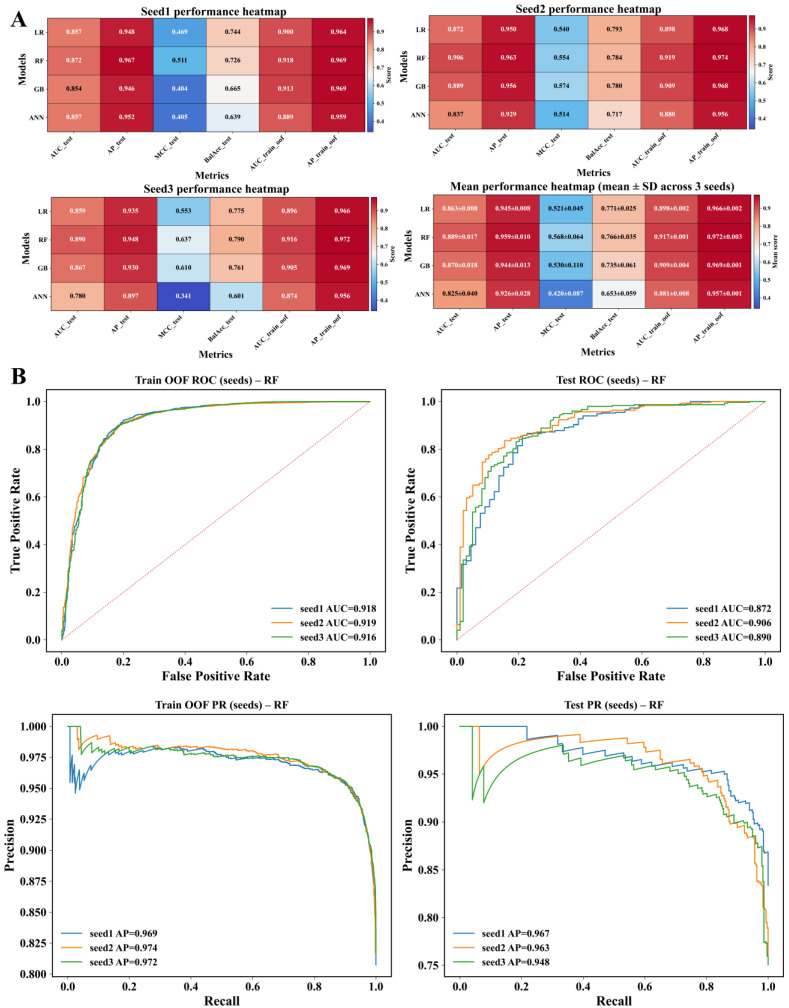
Comparative model performance under scaffold-based evaluation. (**A**) Heatmap summarizing classification performance for logistic regression (LR), random forest (RF), gradient boosting (GB), and artificial neural network (ANN) models. Test-set metrics include area under the receiver operating characteristic curve (AUC), average precision (AP), Matthews correlation coefficient (MCC), and balanced accuracy, together with out-of-fold training AUC and AP. Values represent the mean ± standard deviation across three independent scaffold split seeds. (**B**) Receiver operating characteristic curves (top) and precision–recall curves (bottom) for the random forest classifier evaluated across three scaffold split seeds. Left panels show out-of-fold training performance and right panels show performance on the held-out test sets. Diagonal dashed lines indicate random classification.

Model performance was evaluated using scaffold-based train/validation/test partitions generated with three independent split seeds. Across splits, all models achieved strong out-of-fold discrimination (AUC range 0.881–0.917; AP range 0.957–0.972) (Figure 3A). On held-out test sets, the random forest classifier demonstrated the highest overall performance, achieving a mean test AUC = 0.889 ± 0.017 and mean test AP = 0.959 ± 0.010 (Figure 3B). Logistic regression and gradient boosting showed comparable discrimination with slightly lower precision, while the artificial neural network exhibited reduced stability across scaffold splits. Threshold-dependent metrics, including Matthews correlation coefficient and balanced accuracy, showed consistent ranking, with the random forest model performing best overall (Figure 3A). Seed-resolved receiver operating characteristic and precision–recall curves confirmed stable performance across independent scaffold splits (Figure 3B; Supplementary Figures S1 and S2).

### 2.4. Structural Interpretation of the Top-Performing Model Through Substructure and Shape Analysis

**Figure 4:**
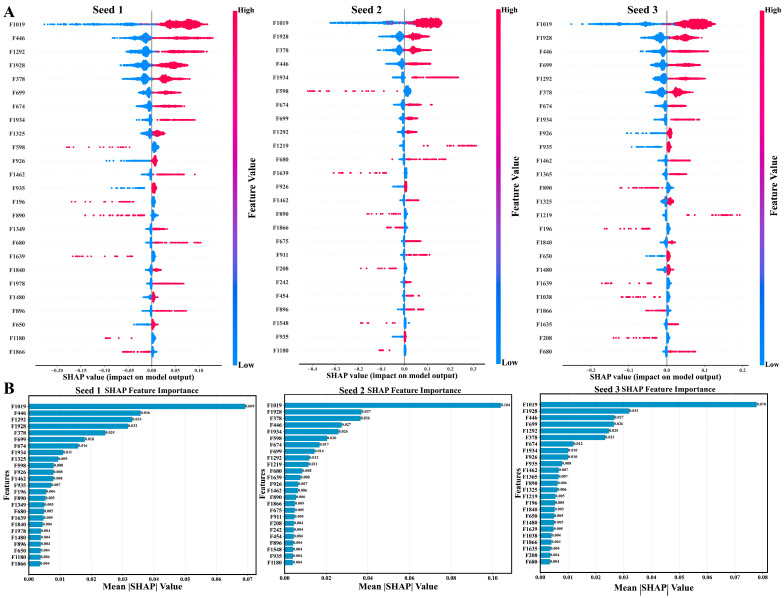
SHAP-based interpretation of the random forest classifier evaluated across three independent scaffold split seeds. (**A**) Shows SHAP beeswarm plots summarizing the distribution of feature contributions to model output, with each point representing an individual compound–feature instance colored by feature value. (**B**) Mean absolute SHAP values for the corresponding features, indicating their relative importance. Across seeds, a consistent set of fingerprint-derived shape features dominates model attribution, demonstrating the stability of learned structural patterns under scaffold-based evaluation.

To examine the structural patterns underlying the predictions of the top-performing classifier, feature attribution analysis was performed on the random forest model using SHAP values. Contributions were evaluated independently across the three scaffold-based split seeds to assess the consistency of substructure-level importance. Across all three seeds, a recurrent set of fingerprint features dominated the attribution profiles (Figure 4A). Several features, including those indexed as F1019, F1928, F446, F378, and F699, consistently ranked among the highest contributors to model output. The relative ordering of these features showed minor seed-dependent variation; however, their overall prominence was preserved, indicating stable reliance on a shared subset of molecular substructures. Visualization of the corresponding circular fingerprint environments revealed that the most influential features mapped to chemically interpretable motifs, including heteroaromatic ring systems, substituted bicyclic cores, and peripheral polar functionalities (Figure 4B). These substructures were distributed across multiple scaffolds present in the dataset and were not confined to a single chemical series, suggesting that the model captured recurring local environments rather than scaffold-specific signatures. To further contextualize these findings, the top thirty substructures ranked by mean SHAP value were extracted and visualized (Supplementary Figure S3A,B). These features encompassed a range of local chemical environments, with varying radii, reflecting contributions from both immediate functional groups and extended substituent patterns. The presence of multiple features associated with the same core motifs but differing radii indicates that both local substitution and broader shape context influenced model predictions. Complementary R-group analysis was conducted on high-scoring compounds to assess substituent-level variability around dominant cores (Supplementary Figure S4). This analysis revealed that structural diversity among the top-ranked molecules was primarily localized to peripheral positions, while core scaffolds remained conserved. Substituent variation frequently involved polar or heteroatom-containing groups, consistent with the substructure importance profiles identified by SHAP analysis. These results demonstrate that the top-performing model relied on a reproducible set of chemically meaningful substructures and shape features across independent scaffold splits. The convergence of feature attribution, substructure visualization, and R-group analysis supports the internal consistency of the learned structure–activity relationships without invoking explicit mechanistic assumptions. To further resolve substituent-level effects, pairwise combinations of R1 and R2 groups were evaluated by aggregating mean screening scores across high-scoring compounds. A heatmap summarizing the mean predicted scores for R1 × R2 combinations observed in at least three compounds is provided in Supplementary Figure S5. This analysis highlights specific substituent pairings associated with elevated mean screening scores while excluding sparsely represented combinations.

### 2.5. Performance Stratification by Scaffold Frequency and Calibration Analysis

To further examine model behavior under varying degrees of structural familiarity, performance of the top-performing random forest classifier was stratified by scaffold frequency observed in the combined training and validation sets. Test-set discrimination metrics were evaluated across scaffold frequency bins, defined by the number of occurrences of each Bemis–Murcko scaffold in the training and validation data. Mean area under the receiver operating characteristic curve, average precision, and Matthews correlation coefficient varied across frequency strata, reflecting differential performance for scaffolds represented at different frequencies in the training distribution (Supplementary Figure S6). Calibration of the predicted probabilities was evaluated by comparing the mean predicted probabilities with observed positive fractions for both out-of-fold training predictions and held-out test predictions, averaged across scaffold split seeds. The resulting calibration curves closely followed the diagonal reference for perfect calibration, with reported Brier scores indicating comparable calibration behavior between the training and test predictions (Supplementary Figure S7). Corresponding scaffold frequency–stratified performance trends for gradient boosting, artificial neural network, and logistic regression models are reported in Supplementary Figure S8, showing analogous variation in discrimination and threshold-dependent metrics across scaffold frequency bins.

### 2.6. Screening and Prioritization of Food-Derived Compounds

**Figure 5:**
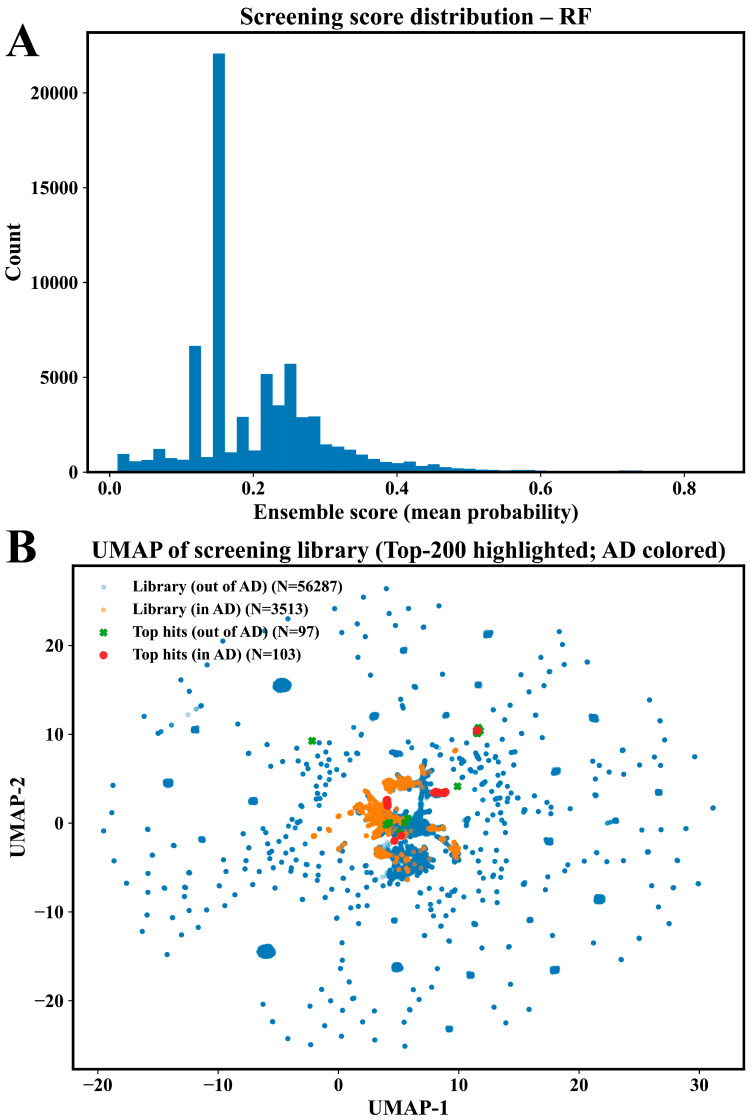
Large-scale screening of the food-derived compound library using the top-performing model. (**A**) Distribution of ensemble-averaged screening scores predicted by the random forest classifier for 69,574 clean food-derived compounds, where scores represent the mean predicted interaction probabilities across three independent random seeds. (**B**) UMAP projection of the screened food-derived library colored by applicability domain membership, with the top 200 highest-scoring compounds highlighted. Points indicate compounds classified as within or outside the applicability domain, illustrating the distribution of high-ranking predictions relative to the chemical space defined by the training data.

Following model selection and validation, the top-performing random forest classifier was applied to a curated food-derived chemical library assembled from FooDB. After standardization and filtering, the final screening library comprised 69,574 clean compounds, all of which satisfied the same preprocessing criteria used for model training (Supplementary Table S4). Predicted interaction probabilities were generated for the full library using an ensemble of models trained across three independent random seeds, and the resulting predictions were averaged to obtain a final screening score for each compound. The resulting screening score distribution showed a strong skew toward lower predicted probabilities, with a progressively decreasing tail extending toward higher scores (Figure 5A). To assess the relationship between predicted hits and the applicability domain of the model, the screened compounds were projected into a low-dimensional chemical space using UMAP and colored by applicability domain membership (Figure 5B). The screening library contained 56,287 compounds outside the applicability domain and 3513 compounds within the applicability domain. Among the top 200 highest-scoring compounds, 97 were located outside the applicability domain and 103 were located within the applicability domain. The complete set of screening predictions for all 69,574 compounds, including ensemble-averaged scores across three seeds and applicability domain assignments, is provided in Supplementary Table S4. The top 200 highest-scoring compounds, ranked by mean screening score, are reported separately in Supplementary Table S5.

### 2.7. Selection of the Reference Crystal Structure and Validation of the Docking Protocol

**Figure 6:**
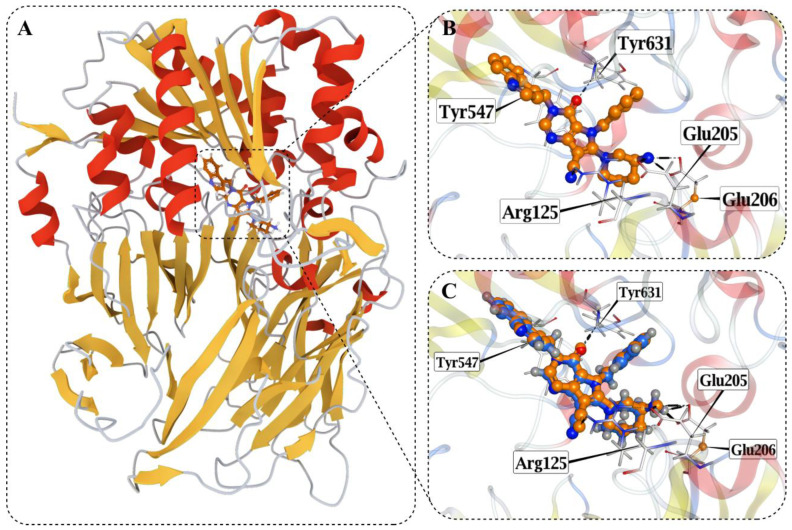
Crystal structure of human DPP4 and validation of the docking protocol. (**A**) Three-dimensional structure of human dipeptidyl peptidase-4 (PDB ID: 4A5S) shown as a cartoon representation, with α-helices shown in red, β-sheets in yellow, and loops in gray. The co-crystallized ligand is shown in stick representation with carbon atoms in orange, nitrogen in blue, and oxygen in red. (**B**) Binding-site view highlighting interactions between the co-crystallized ligand and key residues, including Glu205, Glu206, Arg125, Tyr547, and Tyr631, illustrating hydrogen-bonding and ionic contacts. (**C**) Superposition of the co-crystallized ligand (orange sticks) and the redocked pose (blue sticks), showing close agreement between the experimental and predicted binding modes with an RMSD of 0.327 Å.

Prior to molecular docking, the crystal structure of human dipeptidyl peptidase-4 was selected based on resolution, completeness, and availability of a co-crystallized inhibitor. The structure PDB ID: 4A5S represents human DPP4 crystallized in complex with a heterocyclic DPP4 inhibitor and was determined by X-ray diffraction at a resolution of 1.62 Å (Figure 6A). Analysis of the co-crystallized ligand interactions revealed a well-defined binding mode stabilized by a combination of hydrogen bonding and ionic interactions within the active site (Figure 6B). The ligand formed hydrogen-donor interactions with Glu205 and Glu206, hydrogen-acceptor interactions with Arg125 and Tyr631, and ionic interactions with Glu205 and Glu206, indicating cooperative electrostatic and hydrogen-bonding contributions to ligand stabilization within the catalytic pocket. To validate the docking protocol, the co-crystallized ligand was re-docked into the DPP4 active site (Figure 6C). The redocked pose reproduced the experimental binding orientation with a root-mean-square deviation of 0.327 Å relative to the crystallographic ligand position, confirming the reliability of the docking setup. Docking results for the top 200 screened compounds against the DPP4 binding site are reported in Supplementary Table S6.

### 2.8. Prioritization of Food-Derived Compounds Based on Predicted Interaction Propensity

From the screened food-derived compound library, a subset of high-ranking candidates was prioritized based on ensemble screening scores and favorable interaction patterns within the DPP4 binding site. Selection was restricted to compounds exhibiting consistently high docking scores and stable binding geometries relative to the reference structure. Seven compounds met these criteria and were retained for detailed structural inspection (Table 1). These candidates displayed docking scores ranging from −13.12 to −12.06 kcal/mol and root-mean-square deviation values between 2.24 and 4.78 Å, indicating reproducible binding poses within the DPP4 catalytic pocket.

**Figure 7:**
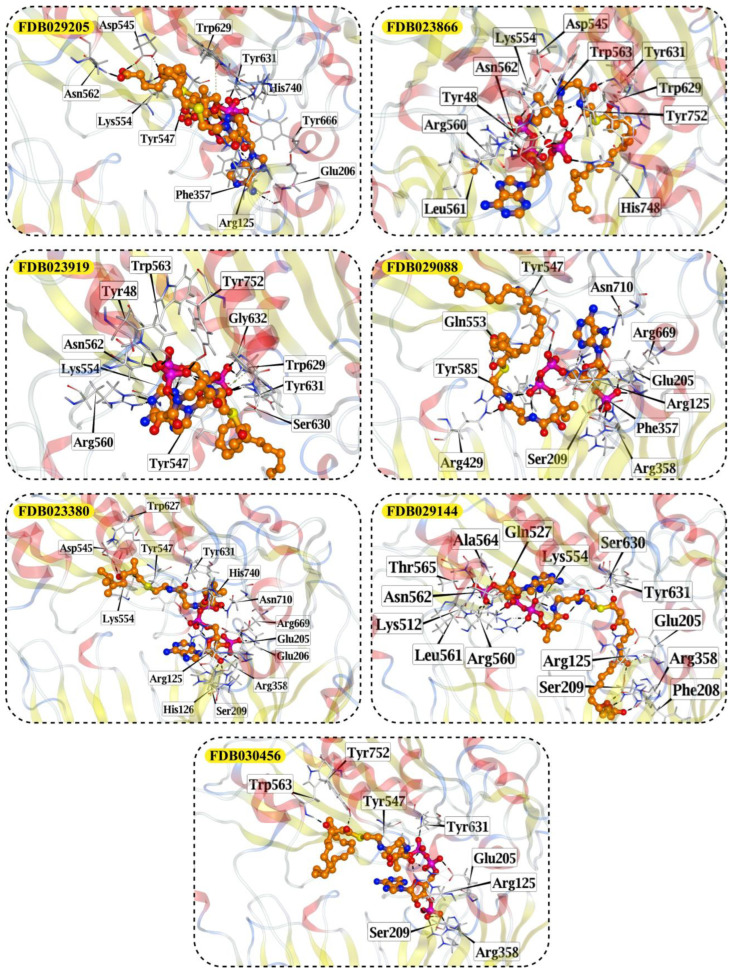
Docked binding poses of the seven selected food-derived compounds (FDB029205, FDB023866, FDB023919, FDB029088, FDB023380, FDB029144, and FDB030456) shown within the active site of human DPP4 (PDB ID: 4A5S).

The prioritized compounds originate from lipid-associated biochemical classes present in food systems, including long-chain acyl–coenzyme A derivatives and oxidized fatty acid conjugates. Their molecular architectures combine extended aliphatic chains with polar head groups, enabling the simultaneous engagement of multiple interaction regions within the DPP4 binding cavity (Figure 7). Although intact coenzyme A-linked derivatives are unlikely to be present in substantial dietary concentrations and may exhibit limited systemic bioavailability, these structures are interpreted as representatives of broader lipid-associated metabolic motifs rather than direct dietary effectors. Their prioritization reflects recurring chemical features compatible with DPP4 interaction propensity rather than presumed physiological exposure to intact CoA conjugates. Comparative interaction analysis relative to the co-crystallized ligand revealed consistent engagement of conserved catalytic and substrate-recognition residues, including Glu205, Glu206, Arg125, Tyr631, Trp629, and Lys554. Interaction profiles were dominated by hydrogen bonding and electrostatic contacts involving phosphate and polar functional groups, complemented by hydrophobic and aromatic interactions stabilizing the aliphatic scaffolds (Figure 7). A complete residue-level interaction summary for all seven compounds is provided in Supplementary Table S7.

### 2.9. Conformational Stability of DPP4 Systems Assessed by Molecular Dynamics Simulations

**Figure 8:**
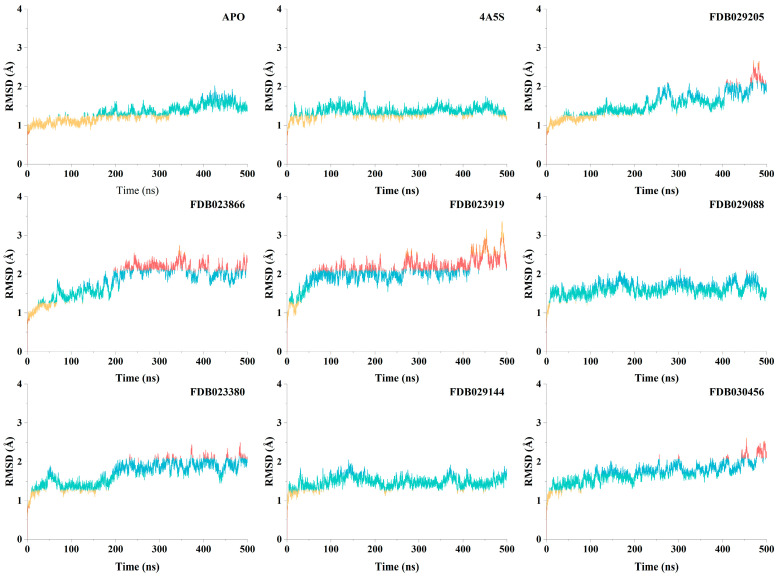
Time evolution of backbone root-mean-square deviation for apo DPP4, the co-crystallized complex (4A5S), and DPP4 complexes with the seven selected food-derived compounds (FDB029205, FDB023866, FDB023919, FDB029088, FDB023380, FDB029144, and FDB030456) over 500 ns of simulation.

To evaluate the structural stability of DPP4 in different binding states, 500 ns molecular dynamics simulations were performed for the apo protein, the co-crystallized complex (4A5S), and the seven selected food-derived compound complexes. Root-mean-square deviation profiles of the protein backbone were monitored over the full simulation period (Figure 8). The apo DPP4 system exhibited a gradual increase in RMSD during the initial phase of the simulation, followed by stabilization at values fluctuating around the 1.4–1.7 Å range for the remainder of the trajectory. After the early equilibration phase, fluctuations remained moderate, indicating preservation of the global protein fold in the absence of a bound ligand. The co-crystallized 4A5S complex showed comparable behavior, with RMSD values stabilizing slightly lower than the apo system and remaining largely within the 1.2–1.5 Å range across the simulation. Fluctuations were limited in amplitude and evenly distributed over time, reflecting the structural consistency of the experimentally resolved binding mode. Among the ligand-bound systems, FDB029205 displayed an initial rise in RMSD followed by stabilization at values primarily within the 1.5–2.0 Å range, with moderate fluctuations observed during the latter portion of the trajectory. These deviations remained bounded, indicating a stable protein–ligand complex. The FDB023866 system reached a stable RMSD plateau after early equilibration, maintaining values predominantly between 1.8 and 2.2 Å, with slightly increased fluctuations relative to the apo and co-crystallized systems but without evidence of progressive drift. For FDB023919, RMSD values increased during the early simulation phase and subsequently fluctuated around 2.0–2.4 Å, with transient excursions toward higher values observed later in the trajectory. Despite these fluctuations, the system did not exhibit sustained instability. The FDB029088 complex showed comparatively lower RMSD values among the ligand-bound systems, stabilizing around 1.4–1.8 Å and maintaining consistent fluctuations throughout the simulation, closely resembling the behavior of the co-crystallized control. The FDB023380 system stabilized after initial equilibration, with RMSD values fluctuating within the 1.6–2.0 Å range. Fluctuation amplitudes remained moderate over time, indicating maintenance of a stable binding environment. For FDB029144, RMSD values remained relatively constrained, stabilizing around 1.4–1.7 Å with limited variability, suggesting a stable protein backbone throughout the simulation. Finally, the FDB030456 complex exhibited RMSD stabilization within the 1.6–2.1 Å range, with gradual increases toward the later stages of the simulation but without abrupt structural deviations. Overall, all ligand-bound systems retained stable backbone conformations over 500 ns, with RMSD values remaining within ranges comparable to or moderately higher than those observed for the apo and co-crystallized controls. These results indicate that binding of the selected food-derived compounds does not induce large-scale destabilization of the DPP4 structure under the simulated conditions.

### 2.10. Residue-Level Flexibility Analysis During Molecular Dynamics Simulations

Residue-wise root-mean-square fluctuation profiles were calculated for apo DPP4, the co-crystallized complex (4A5S), and the seven ligand-bound systems over the 500 ns molecular dynamics simulations (Supplementary Figure S9). For the apo DPP4 system, the majority of residues exhibited low RMSF values across the protein sequence, indicating limited backbone mobility. A pronounced fluctuation peak was consistently observed in a central region of the sequence, approximately spanning residues 180–220, while smaller fluctuations were detected in terminal and loop regions outside this segment. The co-crystallized 4A5S complex displayed a closely similar RMSF profile, with reduced fluctuations across most residues and a dominant peak localized within the same 180–220 residue region, suggesting that this segment represents an intrinsically flexible region of the protein rather than a ligand-induced effect. Among the ligand-bound systems, FDB029205 maintained low RMSF values across the majority of residues, with a single dominant fluctuation peak again centered in the 180–220 residue range, comparable in magnitude and position to that observed in the control systems. The FDB023866 system showed a more pronounced fluctuation within this central region, with elevated RMSF values localized primarily between 190 and 230 residues, while the remainder of the protein exhibited constrained mobility. For FDB023919, RMSF profiles revealed a dominant fluctuation peak in the same central segment, extending approximately from 180 to 220 residues, along with minor fluctuations distributed across other loop regions. The FDB029088 complex exhibited comparatively lower RMSF amplitudes across the sequence, with fluctuations largely confined to the ~180–210 residue region, indicating reduced residue-level mobility relative to several other ligand-bound systems. The FDB023380 system showed elevated fluctuations centered in the 190–230 residue range while maintaining low RMSF values throughout the remaining structured regions of the protein. For FDB029144, residue-level fluctuations remained limited across most of the sequence, with a modest peak observed in the 180–220 residue region, closely resembling the behavior of the co-crystallized complex. Finally, the FDB030456 complex displayed a dominant RMSF peak within the same central region, approximately 180–230 residues, while all other residues exhibited low and stable fluctuation values. Across all systems, residue-level flexibility was consistently localized to a conserved central region of the DPP4 sequence, whereas the remaining residues displayed low RMSF values. This pattern indicates that ligand binding did not introduce new flexible regions but preserved the intrinsic dynamic behavior of the protein backbone.

### 2.11. Protein Compactness During Molecular Dynamics Simulations

To assess the global compactness of DPP4 across the simulated systems, the radius of gyration was monitored over the 500 ns molecular dynamics trajectories for the apo protein, the co-crystallized complex (4A5S), and the seven ligand-bound systems (Supplementary Figure S10). The apo DPP4 system displayed relatively stable radius of gyration values throughout the simulation, with minor fluctuations around a consistent baseline during the first ~400 ns. A gradual increase in Rg was observed toward the final portion of the trajectory, indicating a modest expansion of the protein structure at later simulation times. The co-crystallized 4A5S complex exhibited stable Rg values across the entire 500 ns simulation, with only small-amplitude fluctuations and no pronounced long-term drift, reflecting sustained global compactness in the presence of the co-crystallized ligand. For the FDB029205-bound system, Rg values showed an initial increase during the early phase of the simulation, followed by a plateau and moderate fluctuations across the remaining trajectory. A gradual upward trend was observed toward the later stages, suggesting a slight expansion while maintaining overall structural integrity. The FDB023866 complex demonstrated stable Rg behavior after an initial equilibration period, with fluctuations remaining within a narrow range across the simulation and no abrupt changes indicative of large-scale conformational rearrangements. In the FDB023919 system, Rg values increased during the early portion of the trajectory and stabilized thereafter, with moderate fluctuations persisting throughout the simulation, indicating maintained compactness following initial structural adjustment. The FDB029088-bound complex displayed relatively stable Rg values across the full simulation window, with small-amplitude fluctuations and no evident long-term drift, suggesting the preservation of global protein compactness. For FDB023380, the radius of gyration increased during the first ~200 ns and subsequently stabilized, with moderate fluctuations observed during the latter half of the simulation, indicating a stable yet slightly expanded protein conformation. The FDB029144 system maintained consistent Rg values throughout the simulation, with limited fluctuations and no discernible trends toward expansion or contraction, closely resembling the behavior of the co-crystallized complex. Finally, the FDB030456-bound complex showed a gradual increase in Rg during the simulation, followed by stabilization in the later stages, with fluctuations remaining moderate and comparable to those observed in other ligand-bound systems. Overall, the radius of gyration analysis indicated that all systems retained stable global compactness throughout the 500 ns simulations. Ligand binding did not induce large-scale unfolding or collapse of the DPP4 structure, with observed variations reflecting moderate and system-specific adjustments rather than extensive conformational destabilization.

### 2.12. Collective Motions and Dominant Conformational Variability Revealed by Principal Component Analysis

**Figure 9:**
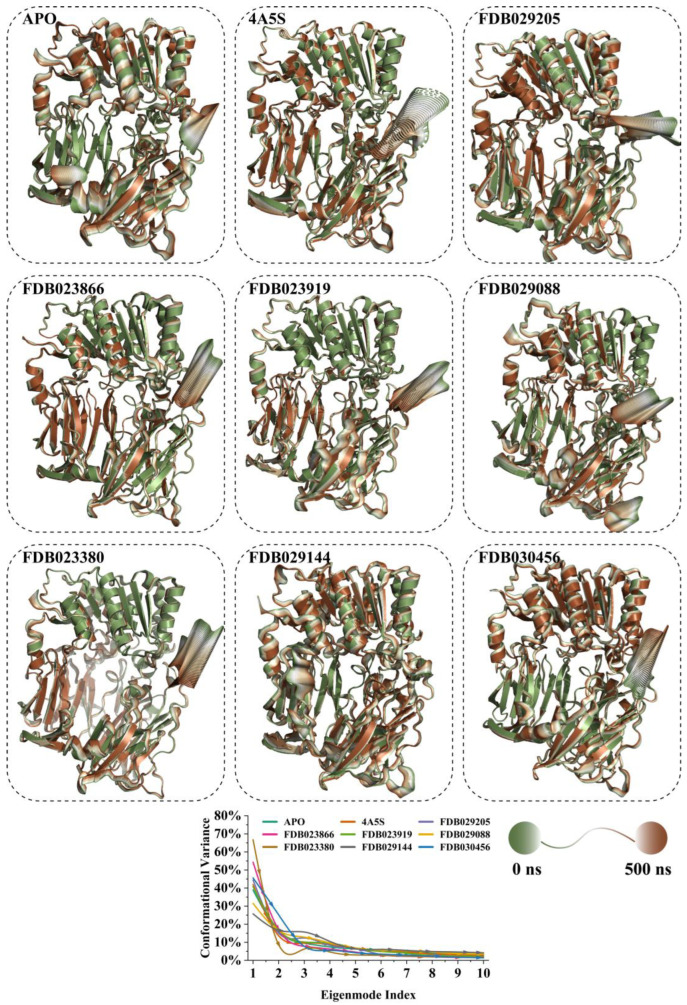
Cartoon representations of the dominant collective motions projected along the first principal component for the apo protein, the co-crystallized complex (4A5S), and the seven ligand-bound systems. Structures are colored to indicate conformational progression from the initial state (0 ns) to the final state (500 ns). The representations highlight large-scale concerted movements of secondary-structure elements captured by the principal component analysis, illustrating system-dependent differences in the amplitude and directionality of dominant motions.

Principal component analysis was performed on the backbone trajectories to characterize the dominant collective motions of DPP4 across the apo system, the co-crystallized complex (4A5S), and the seven ligand-bound systems (Figure 9). The contribution of the first eigenmode to the total conformational variance differed across systems, indicating ligand-dependent modulation of large-scale protein motions. The apo DPP4 system exhibited a first-mode contribution of 38.90%, reflecting moderate intrinsic flexibility. The corresponding cartoon representation showed collective motions distributed across both the α-helical and β-sheet regions, with visible concerted movements of peripheral domains relative to the core structure. For the co-crystallized 4A5S complex, the first eigenmode accounted for 41.90% of the total variance. The dominant motion was characterized by coordinated rearrangements of surface-exposed helices, while the central β-sheet framework remained comparatively constrained, indicating stabilization of the protein architecture in the presence of the co-crystallized ligand. The FDB029205-bound system displayed a first-mode contribution of 44.42%, with enhanced collective motion relative to both the apo and co-crystallized states. Cartoon visualization revealed amplified movements in loop regions and adjacent helices surrounding the binding cavity, while the overall fold remained preserved. In the FDB023866 complex, the first eigenmode contributed 54.29% of the total variance, representing a pronounced concentration of motion within a single dominant mode. The associated structural representation showed extensive concerted movements involving multiple helices and loop segments, indicating increased dynamic coupling across the protein. The FDB023919-bound system exhibited a first-mode contribution of 40.77%, comparable to the co-crystallized complex. The dominant motion involved subtle bending and twisting of peripheral secondary-structure elements, with limited displacement of the protein core. For FDB029088, the first eigenmode accounted for 31.49% of the conformational variance, representing the lowest contribution among the ligand-bound systems except for FDB029144. The corresponding motion was more evenly distributed across higher-order modes, and the cartoon representation showed restrained collective movements with reduced amplitude. The FDB023380 complex displayed the highest first-mode contribution at 66.75%, indicating strong dominance of a single collective motion. Structural visualization revealed large-amplitude concerted movements spanning multiple secondary-structure elements, suggesting enhanced global flexibility relative to other systems. In contrast, the FDB029144-bound system showed a first-mode contribution of 25.71%, the lowest among all systems analyzed. The dominant motion was comparatively subdued, with limited displacement observed in the cartoon representation, indicating a more evenly distributed conformational variance across multiple modes. Finally, the FDB030456 complex exhibited a first-mode contribution of 45.61%, with collective motions involving coordinated shifts of helices and loop regions proximal to the binding site while maintaining overall structural coherence. These results demonstrate that ligand binding modulated the distribution and magnitude of dominant collective motions in DPP4. Differences in first-mode contributions and associated structural movements indicate system-specific effects on protein dynamics without inducing large-scale structural disruption.

To further characterize the conformational landscapes sampled during the simulations, trajectories were projected onto the first two principal components (PC1 and PC2), and conformational states were identified using k-means clustering (Supplementary Figure S11). This analysis revealed distinct clustering patterns and transition pathways across the apo, co-crystallized, and ligand-bound systems. The apo DPP4 system populated three well-separated conformational clusters in PC space, indicating sampling of multiple metastable states. Transitions between clusters occurred progressively along the trajectory, reflecting intrinsic conformational heterogeneity in the absence of a bound ligand. In the co-crystallized 4A5S complex, trajectory points followed a more continuous and curved distribution across PC space. Clustering revealed defined conformational states connected by smooth transitions, indicating restrained yet structured conformational exploration relative to the apo protein. For the FDB029205-bound system, two dominant clusters were observed, separated primarily along PC1. The trajectory exhibited directed transitions between these states, suggesting ligand-associated modulation of dominant motions while maintaining a limited number of preferred conformations. The FDB023866 complex sampled three distinct clusters with pronounced separation along both PC1 and PC2. The distribution indicated broader conformational sampling compared with the co-crystallized complex, consistent with enhanced collective motion observed in the essential dynamics analysis. In the FDB023919-bound system, four clusters were identified, distributed across PC space. Transitions between clusters occurred sequentially, indicating progressive conformational rearrangements rather than abrupt state switching. The FDB029088 system displayed three primary clusters with moderate separation. The trajectory exhibited structured transitions between these states, suggesting constrained but non-static conformational behavior. For FDB023380, two dominant clusters were clearly separated along PC1, with a defined transition pathway between them. This behavior reflected strong dominance of a single collective motion, consistent with the high variance captured by the first principal component. The FDB029144-bound system exhibited three compact clusters with limited spread in PC space. Transitions were localized, indicating restricted conformational exploration relative to other ligand-bound systems. Finally, the FDB030456 complex showed three clusters arranged along a directional path in PC space, with transitions indicating gradual shifts between conformational states over the simulation. The PC1–PC2 projections demonstrated that ligand binding altered the distribution and connectivity of conformational states sampled by DPP4. Differences in clustering patterns and transition pathways reflected system-specific modulation of dominant motions without inducing a loss of structural coherence.

### 2.13. Free Energy Landscape of Dominant Conformational States

**Figure 10:**
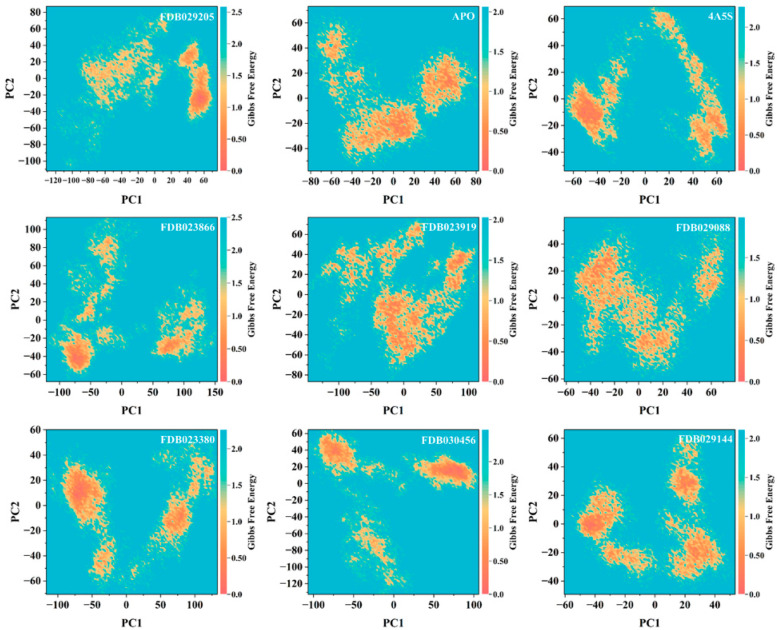
Gibbs free energy landscapes of DPP4 constructed at 300 K by projecting molecular dynamics trajectories onto the first two principal components (PC1 and PC2) for the apo protein, the co-crystallized complex (4A5S), and the seven ligand-bound systems.

To further characterize the thermodynamic features underlying the dominant collective motions, free energy landscapes were constructed by projecting the molecular dynamics trajectories onto the first two principal components (PC1 and PC2) at 300 K (Figure 10). The resulting Gibbs free energy surfaces revealed system-specific distributions of low-energy basins corresponding to preferentially populated conformational states. The apo DPP4 system exhibited multiple low-energy regions distributed across the PC space, indicating the presence of several energetically accessible conformational basins. These basins were moderately separated, reflecting intrinsic conformational heterogeneity in the absence of a bound ligand. For the co-crystallized 4A5S complex, the free energy landscape displayed more localized low-energy regions compared with the apo system. The dominant basin was well-defined, with adjacent shallow basins connected through low-energy pathways, consistent with constrained conformational sampling. The FDB029205-bound system showed a distinct organization of energy basins, with multiple localized low-energy regions distributed along PC1. The separation between basins suggests preferential stabilization of discrete conformational states during the simulation. In the FDB023866 complex, the free energy landscape was characterized by several well-separated low-energy basins spanning a broad range of PC1 and PC2 values. This distribution indicates sampling of multiple energetically favorable conformations, consistent with the broader conformational space observed in principal component projections. The FDB023919-bound system exhibited interconnected low-energy regions forming an extended basin across PC space. The continuity of these regions suggests gradual transitions between conformational states rather than confinement to a single dominant minimum. For FDB029088, the free energy surface displayed a limited number of low-energy basins with moderate spatial separation. The basins were relatively compact, indicating restrained conformational variability compared with systems exhibiting broader landscapes. The FDB023380 complex showed pronounced low-energy basins with clear separation, particularly along PC1. These features indicate the dominance of specific conformational states with a reduced population of intermediate regions. In the FDB029144-bound system, multiple shallow low-energy basins were observed, distributed across PC space. The absence of a single sharply defined minimum suggests balanced sampling across several energetically similar conformations. Finally, the FDB030456 complex exhibited discrete low-energy regions separated by higher-energy barriers, indicating the stabilization of distinct conformational states with limited interconversion during the simulation. The free energy landscapes demonstrate that ligand binding reshaped the energetic topology of DPP4 in a system-dependent manner. Differences in the number, localization, and connectivity of low-energy basins reflect modulation of conformational preferences without evidence of large-scale destabilization.

### 2.14. Hydrogen Bond Persistence Analysis

Hydrogen bond occupancy was evaluated over the full simulation trajectories, with interactions exceeding 10% bond lifetime considered persistent. A residue-wise overview across all systems is provided in Supplementary Figure S12, with complete numerical details reported in Supplementary Table S8. The co-crystallized 4A5S complex exhibited persistent hydrogen bonding primarily involving Glu168 and Tyr593, consistent with stabilization within the canonical binding region. Across the prioritized compounds, hydrogen bond persistence patterns varied by system but were generally characterized by a limited set of dominant, long-lived interactions supported by multiple secondary contacts. Several complexes displayed highly stable interactions with catalytic and substrate-recognition residues, including Glu168, Asp507, Tyr509, Tyr593, Arg522, and Hip702, with individual bond lifetimes exceeding 60% in selected systems. Overall, the hydrogen bond persistence profiles indicate system-specific but stable interaction networks, where a small number of strong, sustained contacts are complemented by transient supporting interactions throughout the simulations.

### 2.15. MM-GBSA Binding Free Energy Analysis

To further quantify interaction energetics, MM-GBSA binding free energy calculations were performed on the equilibrated molecular dynamics trajectories for the co-crystallized complex (4A5S) and all ligand-bound systems (Table 2). The total binding free energy (ΔG_TOTAL_) reflected system-specific balances between the gas-phase interaction energies and solvation contributions. For the co-crystallized 4A5S complex, binding was characterized by a strong electrostatic contribution (ΔE_EL_ = −216.86 ± 0.70 kcal/mol) that was partially offset by a large polar solvation penalty (ΔE_GB_ = 234.30 ± 0.58 kcal/mol), resulting in a total binding free energy of −36.38 ± 0.16 kcal/mol. In the FDB029205 complex, van der Waals interactions (ΔE_VDW_ = −64.48 ± 0.57 kcal/mol) and electrostatic interactions (ΔE_EL_ = −40.15 ± 0.61 kcal/mol) contributed to a gas-phase binding free energy (ΔG_GAS_) of −111.33 ± 0.75 kcal/mol. After the inclusion of solvation effects, the resulting total binding free energy was −62.82 ± 0.43 kcal/mol. The FDB023866 system exhibited a substantial electrostatic contribution (ΔE_EL_ = −164.18 ± 1.73 kcal/mol) combined with favorable van der Waals interactions (ΔE_VDW_ = −55.00 ± 0.19 kcal/mol). Despite a positive polar solvation term (ΔE_GB_ = 57.54 ± 2.07 kcal/mol), the total binding free energy reached −170.33 ± 1.34 kcal/mol. For FDB023919, binding free energy was driven by both van der Waals (ΔE_VDW_ = −71.26 ± 0.19 kcal/mol) and electrostatic interactions (ΔE_EL_ = −112.97 ± 0.48 kcal/mol), counterbalanced by a polar solvation contribution of 135.26 ± 0.74 kcal/mol. The resulting total binding free energy was −58.78 ± 0.46 kcal/mol. The FDB029088 complex showed strong nonbonded interactions, with ΔE_VDW_ of −85.13 ± 0.24 kcal/mol and ΔE_EL_ of −101.63 ± 0.36 kcal/mol. These contributions led to a gas-phase binding free energy of −186.76 ± 0.36 kcal/mol, which after solvation correction yielded a total binding free energy of −157.22 ± 0.68 kcal/mol. In the FDB023380 system, both van der Waals (ΔE_VDW_ = −48.27 ± 0.19 kcal/mol) and electrostatic interactions (ΔE_EL_ = −103.48 ± 0.55 kcal/mol) contributed to binding, accompanied by a negative polar solvation term (ΔE_GB_ = −54.55 ± 0.76 kcal/mol). The combined energetic contributions resulted in a total binding free energy of −214.39 ± 0.61 kcal/mol. For FDB029144, the binding free energy reflected contributions from van der Waals interactions (ΔE_VDW_ = −57.39 ± 0.13 kcal/mol) and electrostatics (ΔE_EL_ = −66.90 ± 0.51 kcal/mol), offset by a polar solvation component of 46.60 ± 0.80 kcal/mol, yielding a total binding free energy of −84.88 ± 0.60 kcal/mol. Finally, the FDB030456 complex displayed moderate van der Waals (ΔE_VDW_ = −51.46 ± 0.14 kcal/mol) and electrostatic contributions (ΔE_EL_ = −30.03 ± 0.36 kcal/mol) together with negative polar solvation energy (ΔE_GB_ = −10.78 ± 0.59 kcal/mol), resulting in a total binding free energy of −99.17 ± 0.57 kcal/mol.

## 3. Discussion

DPP4 plays a central role in postprandial metabolic regulation by modulating the activity of incretin hormones in response to food intake [25]. Here, we integrated machine learning-based classification with structure-guided analysis to prioritize food-derived compounds with the predicted interaction propensity toward DPP4, an enzyme central to postprandial hormonal regulation. Using a chemically diverse reference dataset curated from ChEMBL, models were trained and evaluated under scaffold-based partitioning to reduce structural redundancy between training and test sets. Under this stringent evaluation, the top-performing random forest classifier achieved stable and reproducible discrimination, supporting its suitability for large-scale hypothesis generation across heterogeneous chemical space. Application of this model to a curated food-derived library enabled the prioritization of candidate dietary bioactives, which were subsequently examined using docking and molecular dynamics simulations to assess interaction consistency and structural compatibility. Together, these results demonstrate that computational models can be used to organize the complexity of food chemical space and guide downstream mechanistic exploration of diet–enzyme interactions. Model performance under scaffold-based evaluation provides critical context for interpreting these findings. Unlike random splitting, scaffold-based partitioning prevents closely related chemical series from being shared between training and test sets, thereby imposing a more conservative and chemically realistic assessment of generalization [26]. Across three independent scaffold split seeds, the random forest model consistently achieved high test-set discrimination and precision, indicating that predictions were driven by transferable local chemical environments rather than the memorization of specific scaffolds. This behavior is further supported by the overlap observed between interaction-positive and interaction-negative compounds in global physicochemical space, where no single descriptor and threshold could separate the two classes. Instead, predictive performance emerged from combinations of substructural and shape-related features distributed across chemically distinct scaffolds. In nutritional biochemistry, this is particularly relevant, as food-derived compounds span a broad and continuous spectrum of molecular properties rather than discrete drug-like classes [27]. The ability of the model to retain performance under scaffold separation therefore supports its use as a prioritization tool for chemically novel dietary compounds, enabling the efficient narrowing of large food chemical libraries prior to structure-based analysis and experimental follow-up [28].

Interpretation of the top-performing classifier revealed that predictive performance was driven by a consistent set of substructural features rather than scaffold-specific signatures. Across all three scaffold split seeds, the highest-contributing fingerprint features mapped predominantly to heteroaromatic ring systems combined with peripheral polar functionalities, including hydrogen bond-accepting heteroatoms and substituted aromatic environments. These features recurred across chemically distinct scaffolds, indicating that the model learned transferable local chemical environments associated with interaction propensity rather than relying on global molecular similarity. The stability of feature attribution across independent splits further supports this interpretation, as the relative importance of dominant features showed only a minor variation between seeds. Such convergence suggests that interaction-relevant information is embedded in recurring local motifs that transcend individual chemical series. This observation is consistent with prior computational analyses of DPP4, which frequently report the importance of electrostatic complementarity and hydrogen-bonding capacity within the binding pocket [29]. However, in the present work, these patterns emerged from data-driven attribution rather than predefined interaction rules, supporting their relevance without implying a specific inhibitory mechanism [30]. Application of the validated model to the food-derived chemical library revealed important constraints on interpretability imposed by chemical space coverage. Of the 69,574 screened compounds, the majority fell outside the model’s defined applicability domain, reflecting the substantial structural divergence between food-associated chemistry and the reference interaction dataset. Notably, approximately half of the top 200 highest-scoring compounds were located within the applicability domain, indicating that high-ranking predictions were not exclusively driven by extrapolation beyond the training space. Compounds scoring highly outside the domain should therefore be interpreted as signals warranting caution rather than definitive interaction candidates. This distinction is particularly important at the scale of the present screening effort, which substantially exceeds the size of food-derived libraries typically examined in structure-based studies. Whereas many natural-product docking workflows focus on hundreds to a few thousand compounds, the present analysis required explicit domain control to contextualize predictions across a chemically expansive library [31]. These results underscore the need to pair large-scale nutritional screening with applicability-aware interpretation to avoid the overextension of computational predictions.

Structure-based analysis provided an additional layer of validation by situating high-ranking compounds within the known DPP4 binding environment [32]. Redocking of the co-crystallized ligand reproduced the experimental pose with low deviation, confirming the suitability of the docking protocol for comparative analysis. The seven prioritized food-derived compounds exhibited favorable docking scores and reproducible binding geometries, occupying the canonical catalytic pocket without inducing steric clashes or aberrant orientations. Inspection of residue-level interactions revealed recurrent engagement of Glu205, Glu206, Arg125, and Tyr631, residues that are well-established as central contributors to ligand accommodation within DPP4. These contacts largely overlapped with interaction patterns observed for the reference ligand, suggesting that prioritized compounds align with established binding-site architecture rather than exploiting alternative or artificial interaction modes. Similar residue involvement has been reported in docking studies of both pharmaceutical ligands and natural compounds interacting with DPP4, where acidic residues and aromatic pocket contacts repeatedly emerge as stabilizing elements [33,34]. In this context, docking serves not as proof of functional modulation, but as a structural plausibility filter that supports the consistency of model-based prioritization. Molecular dynamics simulations were used to examine whether the docked poses of prioritized compounds were maintained over time and whether ligand association perturbed the overall structural integrity of DPP4. Across all simulated systems, including the apo protein, the co-crystallized complex, and ligand-bound states, backbone RMSD values remained bounded over 500 ns, with no evidence of unfolding or large-scale destabilization. Ligand-bound systems generally exhibited slightly higher fluctuations than the co-crystallized control, but deviations remained within a narrow and stable range. These observations indicate that the predicted binding modes persist under dynamic conditions and are unlikely to be artifacts of static docking snapshots. Many previous DPP4 docking and molecular dynamics studies relied on simulation windows of approximately 100 ns and interpreted RMSD stability as support for pose plausibility [35]. In this context, the extended simulation times employed here reinforce the consistency of the observed binding configurations without implying enhanced affinity or functional modulation.

Analysis of hydrogen-bond persistence provided further insight into interaction stability at the residue level. Using a conservative occupancy threshold of greater than 10%, each ligand-bound system displayed a limited set of dominant, long-lived hydrogen bonds supported by multiple transient contacts. Notably, specific residues exhibited high persistence in individual complexes, including Glu168 in the FDB029088 system, Hip702 in the FDB029205 complex, and Tyr714 in the FDB023919 system, indicating compact and system-specific interaction networks. Despite this variability, several residues recurrently contributed across systems, reflecting the engagement of conserved regions within the DPP4 binding environment. This pattern is consistent with prior docking and molecular dynamics studies of DPP4, including peptide-focused investigations, which frequently highlight residues such as Tyr547, Ser630, Arg125, and Glu205 as stabilizing interaction partners [33]. The present findings align with this broader residue-level landscape while extending it to chemically distinct, lipid-associated food-derived compounds. MM-GBSA calculations were used to compare relative interaction energetics across systems rather than to infer absolute binding strength. The co-crystallized complex exhibited a moderate total binding free energy, providing a reference point for interpretation. Among the prioritized food-derived compounds, calculated total free energies varied substantially, with some systems displaying more favorable values driven by strong electrostatic contributions, while others reflected a balance between van der Waals interactions and solvation effects. These differences highlight heterogeneity in interaction modes rather than a uniform energetic profile. As is widely recognized, MM-GBSA estimates are sensitive to force-field parameters, solvation models, and trajectory sampling, and therefore should be interpreted qualitatively. Consistent with established practice in DPP4 computational studies, the energetic analysis here serves as supportive evidence that complements docking and dynamics results, rather than as a quantitative predictor of nutritional or biological impact [12].

DPP4 functions as a key enzymatic checkpoint in postprandial metabolism through its role in regulating incretin hormone activity following food intake [16]. From a nutritional perspective, this positions DPP4 as a plausible point of interaction for dietary compounds that may subtly influence metabolic signaling. Previous studies have reported DPP4-associated activity for food-derived peptides and selected polyphenols, supporting the broader concept that components of the diet can engage this enzyme at the molecular level [33]. The present work extends this framework by prioritizing small-molecule food chemistry at scale, rather than focusing on individual compound classes and experimentally preselected candidates. By combining machine learning with structure-based analysis, the study provides a systematic approach for organizing food chemical diversity in relation to a nutritionally relevant enzymatic target, without advancing claims regarding physiological efficacy or health outcomes.

## 4. Materials and Methods

### 4.1. ChEMBL Bioactivity Curation for DPP4 Interaction Potential Modeling

Bioactivity records associated with DPP4 were compiled from ChEMBL version 36, accessed as a locally hosted SQLite database. The objective of this step was not to infer pharmacological inhibition, but to assemble a structured reference set describing interaction-relevant chemical features associated with DPP4. Target-specific records were identified using the ChEMBL target identifier CHEMBL284. Activity annotations were restricted to entries reporting quantitative concentration-based measurements and annotated with defined response types, including IC_50_, K_i_, K_d_, and EC_50_. Only records expressed in nanomolar and micromolar units were retained. To ensure assay-level reliability, only measurements derived from assays with a confidence score of at least 7 were included. Activity entries lacking a numeric standardized value were excluded. Measurements were retained when the standardization flag indicated validated values or when the flag was unassigned, consistent with common ChEMBL curation practice. Retrieved records were annotated with molecular identifiers, canonical SMILES representations where available, assay metadata, and source publication year. An initial extraction yielded 6344 activity entries. Subsequent data refinement was applied to generate a harmonized modeling-ready dataset. Records were retained only when the reported activity relationship was exact, non-positive values were removed, and compounds lacking canonical SMILES were excluded. Following this filtering, 5516 unique compound–activity records remained and were used for all downstream analyses. Concentration values reported in micromolar units were converted to nanomolar units to ensure numerical consistency across the dataset.

### 4.2. Post-Processing of DPP4 Interaction Annotations and Physicochemical Characterization

Activity records retained after initial curation were further processed to generate compound-level interaction annotations suitable for quantitative modeling. Only entries reporting IC_50_ measurements with exact relations were considered at this stage. Reported concentration values expressed in nanomolar or micromolar units were standardized to nanomolar units and transformed to a logarithmic interaction scale (pActivity) by conversion to molar units followed by negative base-10 logarithm. Records yielding undefined or non-positive transformed values were excluded. For compounds associated with multiple activity records, measurements were aggregated at the compound level using a predefined strategy. By default, the highest pActivity value per compound was retained, reflecting the most favorable reported interaction under comparable conditions. Alternative aggregation strategies, including mean or median pActivity, were evaluated but not used for the primary dataset. Compound-level interaction labels were assigned using a threshold of pActivity ≥ 6.0, with entries meeting or exceeding this threshold classified as interaction-positive and remaining entries classified as interaction-negative. A pActivity threshold of 6.0 (corresponding to ≤1 µM activity) was selected because submicromolar potency is commonly considered indicative of meaningful biochemical engagement in DPP4 assays and is frequently applied as a practical boundary in structure–activity modeling studies. Exploratory evaluation of nearby cutoffs yielded comparable structural trends and class distributions, supporting the robustness of this selection. Aggregation and labeling were performed after transformation to ensure consistency across units and assays. Canonical SMILES representations were used to compute physicochemical descriptors with RDKit [36]. Calculated properties included molecular weight, calculated octanol–water partition coefficient, number of hydrogen bond donors and acceptors, topological polar surface area, number of rotatable bonds, and ring count. Lipinski rule-of-five violations [37] were recorded as summary indicators without applying exclusion criteria. Compounds for which valid molecular descriptors could not be computed were removed from subsequent analyses. The final processed tables comprised row-level activity annotations with transformed interaction values, compound-level aggregated interaction metrics, and compound-level physicochemical descriptors.

### 4.3. Dataset Integrity Auditing and Canonicalization

An immutable copy of the input compound table was retained to preserve provenance prior to structural processing. Each SMILES entry was re-evaluated using RDKit sanitization to verify internal chemical consistency. Records that could not be converted into a valid molecular graph were excluded, and the corresponding row indices and failure categories were recorded in a separate structure audit file. For all valid structures, isomeric canonical SMILES were generated and used as the sole structural identifier for downstream exports. This identifier was applied exclusively for record tracking and did not alter previously assigned interaction metrics, interaction labels, or physicochemical descriptors. A final non-overlapping deduplication was performed at the canonical-structure level to remove residual redundancy introduced during prior processing steps. When multiple records mapped to the same canonical structure, interaction annotations were retained using a fixed rule set without recalculation. Continuous interaction values were summarized by retaining the maximum pIC_50_ per structure, binary interaction labels were propagated conservatively so that any interaction-positive record yielded an interaction-positive structure, and auxiliary numeric fields were summarized by the median. Non-numeric annotations were inherited from a representative record. The number of contributing source records per canonical structure was retained as a metadata field. Outputs generated at this stage included a structure-valid table, a canonical structure-level table intended for downstream modeling, a log of excluded structures, and a machine-readable audit report summarizing record counts and aggregation settings.

### 4.4. Scaffold-Based Data Splitting

Model evaluation used scaffold-based partitioning to reduce structural overlap between the training and held-out sets. The deduplicated structure table was used as input, and split membership was assigned at the level of Bemis–Murcko scaffolds [38] computed from the canonical SMILES representation. Scaffolds were generated with RDKit by extracting the Murcko framework for each molecule and encoding the resulting scaffold as a SMILES string. Molecules for which a scaffold could not be generated were assigned an empty scaffold label and treated as a separate group. All molecules sharing an identical scaffold were allocated to the same subset. Scaffold groups were ranked by size and randomly permuted under a fixed pseudorandom seed. Groups were then assigned sequentially to training, validation, and test sets using target fractions of 0.8, 0.1, and 0.1, respectively, without splitting any scaffold group across subsets. This procedure was repeated using three independent seeds (1, 2, and 3) to assess the stability of performance with respect to partitioning. For each seed, a split table was exported containing all original compound annotations together with split membership (train, val, and test) and the seed identifier. File-level checksums (SHA-256) were recorded for the deduplicated input and each split output to support reproducibility. When binary interaction labels were present, the number of interaction-positive compounds per subset was summarized for each seed.

### 4.5. Molecular Representation and Model Training

Morgan fingerprints [39] were used as fixed-length molecular representations. Fingerprints were generated from canonical SMILES using RDKit with radius 2 and 2048 bits. Chirality information was not included unless otherwise stated. Fingerprints were computed once for the full deduplicated dataset and cached for reuse across split seeds, preserving identical feature encoding across all model fits. Training and evaluation were conducted using scaffold-based splits (seeds 1–3). For each seed, the training and validation subsets were combined for model selection, and the test subset was held out and not used during hyperparameter optimization. Hyperparameter tuning used stratified 5-fold cross-validation implemented with RandomizedSearchCV. The optimization objective was the mean cross-validated ROC–AUC, and each randomized search evaluated 25 sampled configurations per model. All randomized procedures, including cross-validation shuffling and parameter sampling, were seeded using the split seed. Parallel execution used all available CPU cores supported by the estimator.

### 4.6. Models and Hyperparameter Search Spaces

Supervised classification models were trained on Morgan fingerprint representations using multiple algorithmic families, including random forest, logistic regression, multilayer perceptron neural networks, gradient boosting, and, where available, extreme gradient boosting. Model selection was performed independently for each scaffold-based split using randomized hyperparameter optimization restricted to the combined training and validation subsets. Hyperparameters were optimized using RandomizedSearchCV with stratified fivefold cross-validation and a fixed random seed. For each model, 25 parameter combinations were sampled, and mean cross-validated ROC–AUC was used as the optimization criterion. Random forest models [40] were tuned with respect to ensemble size, tree depth, node splitting constraints, and feature subsampling. Logistic regression models [41] were optimized over regularization strength and penalty type using standardized fingerprint inputs. Neural network models [42] were tuned over network architecture, activation function, batch size, learning rate, and L2 regularization. Gradient boosting models [43] were optimized over the number of boosting stages, learning rate, and tree depth. Extreme gradient boosting models [44], when used, were tuned over boosting depth, learning rate, subsampling ratios, and regularization parameters. The hyperparameter configuration achieving the highest mean cross-validated ROC–AUC was selected for each model and seed. Using the selected configuration, out-of-fold predictions were generated on the training and validation data, followed by refitting on the full training and validation set and a single evaluation on the held-out test set.

### 4.7. Model Selection, Prediction, and Performance Assessment

For each model and scaffold split, hyperparameter selection was based on the configuration achieving the highest mean ROC–AUC under stratified fivefold cross-validation applied to the combined training and validation data. Using the selected configuration, out-of-fold probability estimates were generated across the training and validation set by refitting the model within each cross-validation fold and predicting only samples not used for fitting. The selected model was subsequently refit on the full training and validation data and evaluated once on the held-out test set, which was not used during model selection or tuning. Discriminative performance was quantified using ROC–AUC and average precision for both out-of-fold training and validation predictions and test predictions. For threshold-dependent evaluation, predicted probabilities were converted to binary class assignments using a fixed threshold of 0.5, and Matthews correlation coefficient [45], balanced accuracy, and confusion matrices were computed on the test set. Receiver operating characteristic and precision–recall curves were generated for each model and split, with out-of-fold training and validation predictions and test predictions overlaid within the same panel.

### 4.8. Aggregation Across Scaffold Split Seeds

Performance metrics were aggregated across the three scaffold split seeds by collating per-seed model evaluation outputs. For each model, per-seed summary tables were merged into a single long-format table containing seed identifiers and all reported metrics. Model-level summary statistics were then calculated across seeds, reporting the mean and standard deviation for ROC–AUC and average precision on the test set and on out-of-fold training and validation predictions, together with threshold-dependent test metrics (Matthews correlation coefficient and balanced accuracy). Models were ranked using the mean test ROC–AUC across seeds as the primary criterion, with mean test average precision used as a secondary criterion when required. Seed-specific prediction files were used to generate per-model overlays of receiver operating characteristic and precision–recall curves, plotted separately for the test set and for out-of-fold training and validation predictions. For each model, receiver operating characteristic and precision–recall analyses were performed across all scaffold split seeds, and performance was summarized using area under the curve and average precision metrics.

### 4.9. Applicability Domain Analysis by Fingerprint Similarity

Applicability domain membership for test-set compounds was assessed using fingerprint similarity to the corresponding training and validation set for each scaffold split seed. Morgan fingerprints used for model development were reused without modification. Pairwise similarity was quantified using the Tanimoto coefficient [46] computed between each test compound fingerprint and all fingerprints in the combined training and validation set. For each test compound, two similarity summaries were calculated: (i) the maximum Tanimoto similarity to any training or validation compound and (ii) the mean similarity across the top five most similar training and validation compounds. Applicability domain membership was defined using a fixed threshold applied to the maximum similarity; compounds with maximum similarity ≥ 0.35 were classified as in-domain, and remaining compounds were classified as out-of-domain. Model performance was then stratified by applicability domain membership using the previously generated test-set probability predictions for each model and seed. Discrimination was summarized separately for in-domain and out-of-domain subsets using ROC–AUC and average precision when both classes were present. Threshold-dependent performance was evaluated using a fixed probability threshold of 0.5 and reported as the Matthews correlation coefficient and balanced accuracy for each subset. Summary statistics were computed per seed and aggregated across seeds by reporting the mean and standard deviation for each metric.

### 4.10. Error Analysis by Similarity, Physicochemical Strata, and Scaffold Frequency

Error patterns were analyzed on the held-out test set by stratifying prediction performance across similarity- and chemistry-defined subsets. Test-set predictions and similarity statistics derived from the applicability domain analysis were used as inputs. For each model and scaffold split seed, test compounds were partitioned by maximum Tanimoto similarity to the training and validation set using fixed similarity bins (0.00–0.20, 0.20–0.35, 0.35–0.50, 0.50–0.70, and 0.70–1.00). A binary domain stratum was defined using the same applicability threshold applied to maximum similarity (in-domain, similarity ≥ 0.35; out-of-domain, similarity < 0.35). Physicochemical slicing was performed using compound descriptors computed previously. When available, molecular weight, LogP, topological polar surface area, hydrogen bond donors, hydrogen bond acceptors, rotatable bonds, and ring count were each discretized into quartiles based on the test-set distribution for the corresponding seed. Prediction-confidence strata were defined by binning predicted probabilities into quartiles. Scaffold-frequency slicing was performed using Bemis–Murcko scaffolds [47] derived from the canonical SMILES. For each seed, scaffold frequency was computed within the combined training and validation subset, and each test compound was assigned the observed count of its scaffold in training and validation. Test compounds were then grouped into frequency bins defined as 0 (unseen scaffold), 1, 2–5, 6–20, and ≥21 occurrences in the training and validation set. Within each stratum, discrimination was summarized using ROC–AUC and average precision when both classes were present. Threshold-dependent performance was computed using a fixed probability threshold of 0.5 and reported as the Matthews correlation coefficient and balanced accuracy. Stratum-wise metrics were calculated per seed and aggregated across seeds by reporting the mean and standard deviation for each model and slice definition.

### 4.11. Model Interpretability Using SHAP

Feature attribution was performed for the top-ranked classifier using Shapley additive explanations (SHAP) [48]. The best-performing model family was selected based on the mean test ROC–AUC aggregated across the three scaffold split seeds. SHAP values were computed independently for each seed using the trained model corresponding to that seed and the Morgan fingerprint feature matrix used for model fitting. For each seed, SHAP analysis was restricted to the combined training and validation subset. A random subset of up to 2000 compounds from training and validation was selected for explanation without replacement, and a background set of up to 200 training and validation compounds was sampled to define the SHAP reference distribution. The explainer type was matched to the estimator class: TreeExplainer was used for tree-based models, LinearExplainer was used for linear models, and DeepExplainer was attempted for neural network models with fallback to KernelExplainer when required. For binary classification, SHAP values were extracted for the interaction-positive class. Global feature importance was summarized using the mean absolute SHAP value across explained samples within each seed. Cross-seed stability of feature attributions was assessed by aggregating feature importances across seeds and reporting the mean and standard deviation of mean absolute SHAP values.

### 4.12. Structural Interpretation and Substituent Analysis

High-importance molecular features were identified from cross-seed SHAP values of the best-performing model. The highest-ranking Morgan fingerprint bit indices were mapped to their corresponding atom-centered environments using the same fingerprint parameters as employed during model training. For each selected bit, compounds activating the feature were identified, and the encoded atomic environments were reconstructed based on the fingerprint radius. These environments were used to delineate recurrent local substructures associated with elevated predicted scores, providing fragment-level attribution of model behavior. Series-level interpretation was conducted on the top-ranked screening candidates by grouping compounds according to shared Murcko scaffolds. The most frequent scaffold among the highest-scoring compounds was selected as the core, subject to a minimum series size requirement. Compounds containing this scaffold were retained, and R-group decomposition was performed using the selected core as the reference. Substituents at each attachment position were assigned, standardized to canonical fragment representations, and summarized by frequency. For each R-group position, mean ensemble prediction scores were calculated per substituent and compared against the series-wide mean, excluding substituents below a minimum occurrence threshold. Representative high-scoring compounds and their corresponding R-group assignments were examined to support the qualitative assessment of scaffold-substituent patterns associated with increased predicted activity.

### 4.13. Calibration Analysis and Decision Threshold Selection

Calibration of predicted probabilities was evaluated using out-of-fold predictions from the combined training and validation set and independently on the held-out test set. Calibration curves were constructed by binning predicted probabilities into ten equal-width intervals over [0, 1] and, within each bin, computing the mean predicted probability and the observed fraction of positive labels. Calibration error was summarized using the Brier score for both out-of-fold training and validation predictions and test predictions. A fixed decision threshold was derived for each seed using only the out-of-fold training and validation predictions. Candidate thresholds were evaluated over the empirical range of predicted probabilities, and the threshold maximizing Matthews correlation coefficient was selected; balanced accuracy was evaluated as an alternative selection criterion. The selected threshold was then applied without modification to the corresponding test-set predictions to obtain threshold-dependent performance estimates. At the selected threshold, Matthews correlation coefficient, balanced accuracy, precision, recall, F1 score, and specificity were computed on the test set. Thresholds and calibration metrics were summarized across seeds by reporting the mean and standard deviation for each model.

### 4.14. Preparation of the Food-Derived Screening Library

A food-derived compound library (FoodDB) [49] was assembled for prospective screening using the trained classifiers. Input records were imported from a delimited text file containing a structural string representation and, when available, compound identifiers and names. Each entry was assigned a stable public identifier. When an external identifier column was provided, its string representation was used; otherwise, identifiers were generated sequentially. Structures were standardized by parsing each input SMILES using RDKit and retaining only entries that could be converted to a valid molecular graph. Canonical isomeric SMILES were generated for all valid structures and used as the internal representation for subsequent screening. When enabled, multi-fragment records were simplified by retaining only the largest fragment by heavy-atom count to mitigate salt and mixture effects. Entries lacking a valid canonical structure were removed. Redundancy within the screening library was removed by deduplicating records on canonical isomeric SMILES, retaining the first occurrence of each unique structure. The refined screening library consisted of three fields per entry: public identifier, compound name, and canonical SMILES.

### 4.15. Fingerprint Generation for Food-Derived Screening Compounds

Molecular fingerprints for the food-derived screening library were generated using the same representation and parameterization applied during model development. Fingerprint settings were read from the training feature configuration and used without modification to ensure feature compatibility between training and screening. Canonical SMILES from the refined screening library were parsed with RDKit and converted to Morgan circular fingerprints (ECFP) as fixed-length binary vectors using the training radius, bit length, and chirality settings. Only molecules that could be parsed into valid RDKit molecular graphs were fingerprinted. For each retained screening entry, the resulting fingerprint vector was paired with the corresponding stable public identifier to preserve unambiguous mapping between model outputs and screening records.

### 4.16. Virtual Screening, Applicability Domain Filtering, and Post-Screening Analysis

A food-derived compound library was curated by canonicalizing SMILES, removing invalid entries, optionally retaining the largest fragment for multicomponent records, and deduplicating by canonical SMILES. Morgan fingerprints were generated for all screening compounds using the identical configuration applied during model training. The best-performing classification model, selected a priori based on mean cross-validated ROC–AUC across seeds, was applied to the screening library using an ensemble strategy in which independently trained models from seeds 1–3 produced per-compound probabilities that were subsequently averaged; the corresponding inter-seed standard deviation was retained as a measure of prediction uncertainty. Compounds were ranked by the ensemble mean probability, and the top-ranked subset was retained for downstream analysis. Applicability domain membership for screening compounds was assessed using fingerprint-based similarity to the training distribution. For each screening compound, the maximum Tanimoto similarity to the combined training and validation set (defined by the scaffold split for seed 1) was computed, and compounds were classified as in-domain when the maximum similarity was at least 0.35. Ranked hits were filtered accordingly to yield an in-domain prioritized set. To characterize chemical space coverage and contextualize prioritized hits, a two-dimensional embedding of the screening library was computed from the fingerprint representation. Prior to embedding, dimensionality was reduced by principal component analysis (up to 50 components, limited by sample size), followed by UMAP with n_neighbors = 25 and min_dist = 0.10. For large libraries, the embedding was computed on a random subsample while enforcing the inclusion of all top-ranked candidates. Redundancy among top-ranked in-domain compounds was reduced by diversity selection using Butina clustering on fingerprint distances (distance = 1 − Tanimoto) with a cutoff of 0.35. From each cluster, the compound with the highest ensemble score was selected as the representative, and the final shortlist was obtained by retaining the top-ranked representatives according to the ensemble screening score.

### 4.17. Protein Structure Selection and Preparation

Atomic coordinates for human dipeptidyl peptidase-4 were obtained from the Protein Data Bank (PDB ID: 4A5S) [50], corresponding to a crystallographic structure resolved in complex with a heterocyclic ligand. The structure was selected based on resolution quality, completeness of the catalytic domain, and clear definition of the active-site architecture. The co-crystallized ligand was removed prior to downstream analyses and was used only to delineate the binding region. Structural preprocessing was carried out using molecular modeling software UCSF ChimeraX, version 1.6 (University of California, San Francisco, CA, USA) [51]. All crystallographic water molecules and non-protein heteroatoms were deleted. Residues with incomplete side-chain coordinates or unresolved regions were identified from the deposited model and reconstructed using restrained comparative modeling to preserve local backbone geometry [52]. Protonation states of ionizable residues were assigned assuming near-physiological conditions (pH 7.4) using structure-based pKa estimation, with manual inspection of residues lining the catalytic pocket to ensure chemically consistent states. Protein termini were modeled in their zwitterionic forms. Hydrogen atoms were added following protonation assignment, and standard protein force-field parameters were applied. The refined structure was subjected to energy minimization with positional restraints on heavy atoms to relieve local steric strain introduced during reconstruction while maintaining the experimentally determined fold using AMBER24 [53]. The resulting minimized protein model was used as the receptor for subsequent molecular docking calculations.

### 4.18. Ligand 3D Preparation for Docking

A ranked list of 200 candidate compounds was exported to a comma-separated file containing compound identifiers and canonical SMILES strings. Ligand three-dimensional coordinates were generated with RDKit. Each SMILES string was parsed into an RDKit molecule object and explicit hydrogen atoms were added prior to conformer generation. Initial 3D conformations were built using the ETKDGv3 [54] embedding procedure with a fixed random seed to ensure reproducibility. Embedded structures were energy-minimized using the MMFF94 force field with an iteration cap of 200 steps. Successfully minimized ligands were written individually to Structure Data File (SDF) format using the compound public identifier as the filename. Molecules failing SMILES parsing, embedding, or MMFF optimization were recorded with row index and failure category and exported to a separate log file. SDF outputs were converted to MOL2 format using Open Babel with protonation states assigned at pH 7.4 [55]. Protonated MOL2 files were subsequently converted to PDBQT format using Open Babel utilities to generate docking-ready inputs. All ligand files were stored using consistent base filenames across formats to preserve traceability between the ranked hit list and downstream docking results.

### 4.19. Molecular Docking

Docking calculations were performed for the prepared set of 200 food-derived compounds using AutoDock Vina (v1.2.5) [56]. The refined human dipeptidyl peptidase-4 structure (PDB ID: 4A5S) served as the receptor model. Prior to docking, the protein was converted to docking-compatible format through the removal of residual solvent molecules, addition of polar hydrogen atoms, assignment of partial atomic charges, and generation of PDBQT coordinates. The docking search space was defined based on the location of the co-crystallized ligand in the reference structure and encompassed the catalytic cavity and adjacent subsites. The docking search space was defined based on the location of the co-crystallized ligand in the reference structure and encompassed the catalytic cavity and adjacent subsites. The grid box was centered at coordinates (center_x = 13.861, center_y = 35.472, center_z = 53.306) with dimensions of size_x = 15.80 Å, size_y = 12.64 Å, and size_z = 12.64 Å (grid spacing 0.316 Å), ensuring complete coverage of the DPP4 active site region. Docking simulations were conducted with an exhaustiveness parameter of 20 to balance sampling depth and computational efficiency. The number of output binding modes was set to 9, and the energy range parameter was set to 3 kcal/mol, consistent with the default AutoDock Vina configuration. No fixed random seed was specified, allowing for stochastic initialization by the Vina engine. Resulting poses were ranked by predicted binding score, and the top-ranked pose for each compound was selected for subsequent analysis. Protein–ligand interaction patterns were examined using automated interaction profiling to identify hydrogen bonding, hydrophobic contacts, and other noncovalent interactions within the catalytic pocket.

### 4.20. Molecular Dynamics Simulations

All-atom molecular dynamics simulations were conducted for human dipeptidyl peptidase-4 in both unbound and ligand-associated states. Simulated systems comprised the ligand-free enzyme and protein–ligand complexes formed with the seven selected food-derived compounds (FDB029205, FDB023866, FDB023919, FDB029088, FDB023380, FDB029144, and FDB030456). In addition, the crystallographic structure corresponding to PDB ID 4A5S was included as a reference complex. Ligand selection was based on combined docking score ranking and qualitative assessment of active-site interaction patterns. Simulations were performed using the c suite with GPU acceleration. Protein atoms were represented using the ff19SB force field [56]. Ligand topologies were generated with GAFF2 parameters [57], and partial atomic charges were assigned using the AM1-BCC scheme [58]. System assembly and parameterization were carried out with LEaP. Each system was solvated in a truncated octahedral box of OPC water molecules, maintaining a minimum solute-to-boundary distance of 12 Å. Counterions were added to neutralize the systems, followed by the addition of monovalent ions to reach an ionic strength of 0.1 M. Covalent bonds involving hydrogen atoms were constrained using the SHAKE algorithm, allowing a time step of 2 fs. Long-range electrostatic interactions were treated with the particle mesh Ewald method, and a 10 Å cutoff was applied to short-range nonbonded interactions. Energy minimization was performed in two sequential stages, beginning with steepest descent optimization (50,000 steps) followed by conjugate gradient (20,000 steps) refinement, with positional restraints applied to protein heavy atoms. Systems were then gradually heated from near 0 K to 300 K under constant-volume conditions using Langevin dynamics. Equilibration was carried out in the isothermal–isobaric ensemble at 300 K and 1 atm. Production simulations were performed for 500 ns under constant pressure and temperature. Trajectory coordinates were recorded at 50 ps intervals for subsequent analyses.

### 4.21. Trajectory Processing and Alignment

Molecular dynamics trajectories were processed using CPPTRAJ [59] within the AMBER24 software package. Periodic boundary conditions were removed prior to analysis. All frames were superimposed onto a reference structure derived from the equilibrated phase using Cα atom alignment to eliminate overall translational and rotational motion.

#### 4.21.1. Structural Stability and Conformational Flexibility

Backbone stability was assessed by calculating Cα root-mean-square deviation values over the full duration of the production simulations relative to the equilibrated reference structure. Residue-level flexibility profiles were obtained by computing Cα root-mean-square fluctuations across all frames. Global compactness of the protein was evaluated by monitoring the radius of gyration throughout each trajectory.

#### 4.21.2. Essential Dynamics and Conformational Sampling

Collective motions were characterized through principal component analysis performed on Cα positional fluctuations. Covariance matrices were constructed from aligned trajectories and diagonalized to extract eigenvectors representing dominant modes of motion. Trajectory projections along the first two principal components were used to examine large-scale conformational sampling. Conformational clustering was performed based on backbone RMSD using a k-means algorithm applied to frames sampled at regular intervals. Representative structures were selected from the most populated clusters according to centroid proximity.

#### 4.21.3. Free Energy Landscape Analysis

Two-dimensional free energy landscapes were generated by mapping trajectory projections onto the first two principal components. Free energy surfaces were calculated at 300 K using the Boltzmann relation, where conformational free energy was derived from normalized probability densities. Energy minima were identified to delineate dominant conformational basins sampled during the simulations.

#### 4.21.4. Protein–Ligand Hydrogen Bond Analysis

Intermolecular hydrogen bonding between the protein and bound ligands was evaluated across production trajectories using geometric criteria. Hydrogen bonds were defined by a donor–acceptor distance threshold of 3.5 Å and a donor–hydrogen–acceptor angle cutoff of 120°. Occupancy and persistence metrics were calculated to distinguish stable interactions from transient contacts within the binding region.

#### 4.21.5. Binding Free Energy Estimation

Binding free energies were evaluated using the Molecular Mechanics-Generalized Born Surface Area method as implemented in the MMPBSA.py [60] module of AMBER24. For MM-GBSA analysis, structural snapshots were extracted from the final 100 ns of each trajectory, corresponding to the equilibrated phase as assessed by stabilization of backbone RMSD and protein–ligand interaction profiles. This yielded 2000 evenly spaced frames per system for binding free energy estimation. The binding free energy was calculated as the difference between the free energy of the protein–ligand complex and the sum of the free energies of the isolated protein and ligand, according to:

(1) $$\Delta G_{bind}={\Delta G}_{R+L}-\left(\Delta G_{R}+{\Delta G}_{L}\right)$$

The total free energy of each state was decomposed into molecular mechanics and solvation components:

(2) $$G=E_{bond}+E_{VDW}+E_{elec}+G_{GB}+G_{SA}-TS_{S}$$

where bonded, van der Waals, and electrostatic terms were obtained from the molecular mechanics force field, polar solvation contributions were estimated using the Generalized Born model, and nonpolar solvation energies were derived from solvent-accessible surface area. Entropic contributions were not included. All reported energy values are expressed in kilocalories per mole.

#### 4.21.6. Data Analysis and Modeling

All data processing, modeling, and analysis were performed using Python v3.10 [61]. Bioactivity data curation and transformation were conducted with pandas (v1.5.3) [62] and NumPy (v1.23.5) [63]. Molecular representation, physicochemical descriptor calculation, Bemis–Murcko scaffold generation, and fingerprint computation were performed using RDKit (v2022.09.5). Machine-learning workflows, including model training, cross-validation, and hyperparameter optimization, were implemented with scikit-learn (v1.2.2) [64]. Extreme gradient boosting models, when used, were trained with XGBoost (v1.7.6) [65]. Scaffold-based data partitioning and fingerprint similarity calculations were carried out using RDKit utilities (v2022.09.5). Dimensionality reduction and chemical space visualization employed principal component analysis from scikit-learn followed by UMAP-learn (v0.5.3) [66]. Model interpretability analyses were performed using SHAP (v0.41.0), with explainer selection matched to estimator class. Visualization and figure generation used Matplotlib (v3.7.1) and Seaborn (v0.12.2) [67]. Random seeds were fixed across all stochastic procedures to ensure reproducibility, and intermediate datasets, split tables, and prediction outputs were retained for downstream validation and auditability.

## 5. Conclusions

DPP4 represents a nutritionally relevant enzymatic interface linking dietary chemical exposure to postprandial metabolic regulation. In this work, we applied an integrated computational framework combining machine learning, structure-based screening, and molecular dynamics simulations to systematically evaluate the interaction potential of dietary and supplemental compounds with DPP4. By training predictive models under scaffold-based evaluation and applying them to a large food-derived chemical library, we identified recurring structural features associated with predicted interaction propensity across chemically diverse compounds. Structure-based analyses placed prioritized compounds within the established DPP4 binding environment and demonstrated stable interaction patterns under dynamic conditions, supporting the internal consistency of the computational prioritization. These findings do not imply pharmacological inhibition or dietary efficacy. Instead, they illustrate how data-driven approaches can be used to organize the chemical diversity of dietary and supplemental compounds and to generate testable hypotheses regarding their potential interactions with nutritionally relevant enzymes. Overall, this study highlights the utility of machine learning-guided screening as a complementary tool in nutritional biochemistry, enabling the systematic prioritization of dietary compounds for focused experimental investigation. Integrating such computational strategies with biochemical and nutritional validation will be essential for advancing our mechanistic understanding of diet–enzyme interactions within complex metabolic systems.

## 6. Limitations and Outlook

While the present study provides a systematic and internally consistent computational assessment of dietary and supplemental compounds in relation to DPP4, experimental validation will be required to further substantiate these predictions. In vitro assays can be used to evaluate whether the prioritized compounds engage DPP4 under controlled biochemical conditions, while in vivo studies may clarify how such interactions are influenced by digestion, metabolism, and physiological exposure. These experimental steps are essential for translating computational prioritization into biological context, but they do not detract from the value of the current framework. Instead, they represent a logical extension of a data-driven screening strategy designed to operate at a scale and chemical diversity that is not readily accessible through experimental approaches alone. An additional methodological consideration concerns the molecular dynamics simulations, where each complex was represented by a single 500 ns trajectory. Although this duration permits substantial conformational sampling, molecular dynamics simulations remain inherently stochastic, and replicate runs could further strengthen statistical confidence. In addition, MM-GBSA energies are interpreted for relative comparison within the dataset rather than as quantitative affinity estimates, particularly for large, flexible ligands where entropic contributions are approximated. By narrowing large dietary chemical spaces to a focused set of structurally plausible candidates, the present work establishes a strong foundation for targeted in vitro and in vivo investigation of diet–enzyme interactions.

## Figures and Tables

**Table 1:** List of seven food-derived compounds selected based on docking score and binding pose stability against DPP4.

FoodDB ID	Common Name	2D Structure	Docking Score (kcal/mol)	RMSD (Å)
FDB029205	CoA-omega-COOH-dinor-LTE4	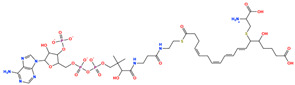	−13.12	3.13
FDB023866	Clupanodonyl CoA	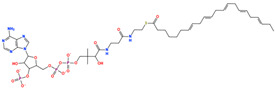	−12.63	2.24
FDB023919	Hexacosanoyl-CoA	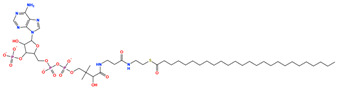	−12.42	2.30
FDB029088	(3S)-Hydroxy-tetracosa-6,9,12,15,18,21-all-cis-hexaenoyl-CoA	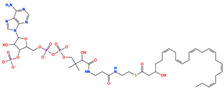	−12.40	4.78
FDB023380	3-Hydroxy-2,6-dimethyl-5-methylene-heptanoyl-CoA	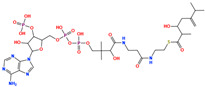	−12.21	2.36
FDB029144	18,20-Dioxo-20-CoA-leukotriene B4	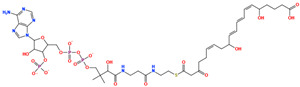	−12.18	2.25
FDB030456	3-Oxo-eicosatrienoyl-CoA	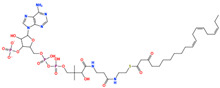	−12.06	2.92

**Table 2:** MM-GBSA binding free energy decomposition for the co-crystallized complex (4A5S) and ligand-bound systems calculated from molecular dynamics trajectories. Reported values include van der Waals energy (ΔE_VDW_), electrostatic energy (ΔE_EL_), polar solvation energy (ΔE_GB_), non-polar solvation energy (ΔE_SASA_), gas-phase binding free energy (ΔG_GAS_), solvation contribution (ΔG_SOLV_), and total binding free energy (ΔG_TOTAL_). Values are shown as the mean ± standard deviation in kcal/mol.

Complex	MM-GBSA Binding Free Energy Analysis (Units: kcal/mol)						
	ΔE_VDW_	ΔE_EL_	ΔE_GB_	ΔE_SASA_	ΔG_GAS_	ΔG_SOLV_	ΔG_TOTAL_
4A5S	−47.94 ± 0.12	−216.86 ± 0.70	234.30 ± 0.58	−5.88 ± 0.07	−264.80 ± 0.68	228.42 ± 0.58	−36.38 ± 0.16
FDB029205	−64.48 ± 0.57	−40.15 ± 0.61	51.24 ± 0.81	−9.42 ± 0.024	−111.33 ± 0.75	48.81 ± 0.69	−62.82 ± 0.43
FDB023866	−55.00 ± 0.19	−164.18 ± 1.73	57.54 ± 2.07	−8.67 ± 0.018	−219.19 ± 1.07	48.86 ± 2.06	−170.33 ± 1.34
FDB023919	−71.26 ± 0.19	−112.97 ± 0.48	135.26 ± 0.74	−9.81 ± 0.021	−184.24 ± 0.52	125.45 ± 0.73	−58.78 ± 0.46
FDB029088	−85.13 ± 0.24	−101.63 ± 0.36	41.22 ± 0.69	−11.67 ± 0.022	−186.76 ± 0.36	29.54 ± 0.70	−157.22 ± 0.68
FDB023380	−48.27 ± 0.19	−103.48 ± 0.55	−54.55 ± 0.76	−8.07 ± 0.014	−151.75 ± 0.52	−62.63 ± 0.75	−214.39 ± 0.61
FDB029144	−57.39 ± 0.13	−66.90 ± 0.51	46.60 ± 0.80	−7.18 ± 0.021	−124.30 ± 0.56	39.42 ± 0.79	−84.88 ± 0.60
FDB030456	−51.46 ± 0.14	−30.03 ± 0.36	−10.78 ± 0.59	−6.89 ± 0.017	−81.49 ± 0.34	−17.68 ± 0.59	−99.17 ± 0.57

ΔE_VDW_: van der Waals energy, ΔE_EL_: electrostatic energy, ΔE_GB_: polar component of the solvation free energy, ΔE_SASA_: non-polar component of the solvation free energy, ΔG_GAS_: gas-phase binding free energy (ΔE_VDW_ + ΔE_EL_), ΔG_SOLV_: solvation contribution to binding free energy, ΔG_TOTAL_: total binding free energy.

## Data Availability

The original contributions presented in this study are included in the article/Supplementary Material. Further inquiries can be directed to the corresponding author.

## Supplementary Materials

The following supporting information can be downloaded at: https://www.mdpi.com/article/10.3390/ph19030349/s1. Figure S1: Receiver operating characteristic curves for logistic regression, random forest, gradient boosting, and artificial neural network models evaluated using three independent scaffold-based splits. For each model, curves are shown for out-of-fold training predictions and for held-out test sets; Figure S2: Precision–recall curves for logistic regression, random forest, gradient boosting, and artificial neural network models evaluated using three independent scaffold-based splits; Figure S3A: Visualization of the thirty highest-ranking fingerprint substructures based on mean SHAP value. Each substructure is annotated by fingerprint bit index and radius, illustrating recurrent local chemical environments and shape features contributing to the random forest model output; Figure S4: R-group analysis of representative high-scoring compounds identified by the random forest model, highlighting conserved core scaffolds and variable peripheral substituents. Predicted scores are reported for each compound, and substituent variation is shown relative to shared cores to illustrate structural diversity among top-ranked predictions; Figure S5: Heatmap showing mean screening scores for observed combinations of R1 and R2 substituents among high-scoring compounds predicted by the top-performing model. Only substituent pairs represented by at least three compounds are shown; combinations with fewer observations were masked. Mean scores are annotated within each cell together with the number of contributing compounds; Figure S6: Mean test-set performance of the random forest classifier evaluated across scaffold frequency bins defined by the number of occurrences of each scaffold in the combined training and validation data. Panels report area under the receiver operating characteristic curve, average precision, and Matthews correlation coefficient, averaged across three independent scaffold split seeds; Figure S7: Calibration curves for the random forest classifier showing the relationship between mean predicted probability and observed positive fraction. Curves are shown for out-of-fold training predictions and for held-out test predictions, averaged across three scaffold split seeds. The dashed diagonal indicates perfect calibration, and corresponding Brier scores are reported in the legend; Figure S8: Mean test-set performance of gradient boosting, artificial neural network, and logistic regression classifiers evaluated across scaffold frequency bins defined by the number of occurrences of each scaffold in the combined training and validation data. Panels report average precision, area under the receiver operating characteristic curve, and Matthews correlation coefficient, averaged across three independent scaffold split seeds; Figure S9: Root-mean-square fluctuation profiles of DPP4 residues for the apo protein, the co-crystallized complex (4A5S), and complexes with the seven selected food-derived compounds over 500 ns of simulation; Figure S10: Radius of gyration (Rg) profiles of DPP4 over 500 ns molecular dynamics simulations for the apo protein, the co-crystallized complex (4A5S), and the seven ligand-bound systems; Figure S11: Two-dimensional projections of the molecular dynamics trajectories onto the first two principal components (PC1 and PC2) for the apo protein, the co-crystallized complex (4A5S), and the seven ligand-bound systems. Each point represents a trajectory frame colored according to k-means clustering. Cluster centroids are indicated and annotated with the corresponding simulation time (ns) at which the centroid structure occurs. Arrows denote transitions between dominant conformational states along the simulation trajectory; Figure S12: Heatmap representation of hydrogen bond lifetime (%) for the co-crystallized complex (4A5S) and all ligand-bound systems over the full simulation trajectories. Values indicate cumulative hydrogen bond occupancy for each residue–compound pair, highlighting persistent interactions and their relative contributions across systems; Table S1: Scaffold-based dataset partitioning using Bemis–Murcko scaffold-based splitting with random seed 1; Table S2: Scaffold-based dataset partitioning using Bemis–Murcko scaffold-based splitting with random seed 2; Table S3: Scaffold-based dataset partitioning using Bemis–Murcko scaffold-based splitting with random seed 3; Table S4: List of 69,574 standardized compounds with canonical molecular representations following preprocessing; Table S5: Top 200 food-derived compounds predicted by the machine learning model, including predicted scores and applicability domain assignments for each compound; Table S6: Docking results for the top-ranked compounds, including binding energies, interaction residues, and root-mean-square deviation values obtained using the validated docking protocol; Table S7: Residue-level interaction fingerprint analysis for the co-crystallized complex and ligand-bound systems, including hydrogen bonding and hydrophobic interaction types relative to the reference crystal structure; Table S8: Detailed residue-level hydrogen bond occupancy analysis for the apo protein, the co-crystallized complex (4A5S), and all ligand-bound systems.

## Abbreviations

The following abbreviations are used in this manuscript:

AD	Applicability Domain
AUC	Area Under the Receiver Operating Characteristic Curve
ANN	Artificial Neural Network
AP	Average Precision
BalAcc	Balanced Accuracy
CoA	Coenzyme A
CPPTRAJ	Coordinate Processing and Trajectory Analysis
DPP4	Dipeptidyl Peptidase-4
ECFP	Extended-Connectivity Fingerprints
EC_50_	Half Maximal Effective Concentration
GB	Gradient Boosting
GBSA	Generalized Born Surface Area
HBD	Hydrogen Bond Donor
HBA	Hydrogen Bond Acceptor
IC_50_	Half Maximal Inhibitory Concentration
K_i_	Inhibition Constant
K_d_	Dissociation Constant
LogP	Octanol–Water Partition Coefficient
LR	Logistic Regression
