# Supplementary material for: Machine Learning Exploration of Food-Derived Chemical Space for Potential Nutritional Metabolic Regulators Targeting Dipeptidyl Peptidase-4
Source: Pharmaceuticals (Basel). 2026 Feb 24;19(3):349. doi: 10.3390/ph19030349 (PMC13029763; doi:10.3390/ph19030349)
Supplement: Supplementary file 1 [file pharmaceuticals-19-00349-s001.zip › pharmaceuticals-4146389-Supplementary Figures.pdf]

Machine Learning Exploration of Food-Derived Chemical Space for Potential Nutritional Metabolic Regulators Targeting Dipeptidyl Peptidase-4

Nada A. Alzunaidy [1,*]

[1] Department of Food Science and Human Nutrition, College of Agriculture and Food, Qassim University, Buraydah 51452, Saudi Arabia.

Corresponding Author: Nada A. Alzunaidy; Department of Food Science and Human Nutrition, College of Agriculture and Food, Qassim University, Buraydah 51452, Saudi Arabia, Email: n.alznedy@qu.edu.sa

Table of contents

Figure S1. Receiver operating characteristic curves for logistic regression, random forest, gradient boosting, and artificial neural network models evaluated using three independent scaffold-based splits. For each model, curves are shown for out-of-fold training predictions and for held-out test sets.. 4

Figure S2. Precision–recall curves for logistic regression, random forest, gradient boosting, and artificial neural network models evaluated using three independent scaffold-based splits.. 5

Figure S3A. Visualization of the thirty highest-ranking fingerprint substructures based on mean SHAP value. Each substructure is annotated by fingerprint bit index and radius, illustrating recurrent local chemical environments and shape features contributing to the random forest model output. 6

Figure S4. R-group analysis of representative high-scoring compounds identified by the random forest model, highlighting conserved core scaffolds and variable peripheral substituents. Predicted scores are reported for each compound, and substituent variation is shown relative to shared cores to illustrate structural diversity among top-ranked predictions.. 8

Figure S6. Heatmap showing mean screening scores for observed combinations of R1 and R2 substituents among high-scoring compounds predicted by the top-performing model. Only substituent pairs represented by at least three compounds are shown; combinations with fewer observations were masked. Mean scores are annotated within each cell together with the number of contributing compounds. 9
Figure S7. Mean test-set performance of the random forest classifier evaluated across scaffold frequency bins defined by the number of occurrences of each scaffold in the combined training and validation data. Panels report area under the receiver operating characteristic curve, average precision, and Matthews correlation coefficient, averaged across three independent scaffold split seeds. 10
Figure S8. Calibration curves for the random forest classifier showing the relationship between mean predicted probability and observed positive fraction. Curves are shown for out-of-fold training predictions and for held-out test predictions, averaged across three scaffold split seeds. The dashed diagonal indicates perfect calibration, and corresponding Brier scores are reported in the legend. 11
Figure S9. Mean test-set performance of gradient boosting, artificial neural network, and logistic regression classifiers evaluated across scaffold frequency bins defined by the number of occurrences of each scaffold in the combined training and validation data. Panels report average precision, area under the receiver operating characteristic curve, and Matthews correlation coefficient, averaged across three independent scaffold split seeds. 12
Figure S10. Root-mean-square fluctuation profiles of DPP4 residues for the apo protein, the co-crystallized complex (4A5S), and complexes with the seven selected food-derived compounds over 500 ns of simulation. 13
Figure 11. Radius of gyration (Rg) profiles of DPP4 over 500 ns molecular dynamics simulations for the apo protein, the co-crystallized complex (4A5S), and the seven ligand-bound systems. 14
Figure 12. Two-dimensional projections of the molecular dynamics trajectories onto the first two principal components (PC1 and PC2) for the apo protein, the co-crystallized complex (4A5S), and the seven ligand-bound systems. Each point represents a trajectory frame colored according to k-means clustering. Cluster centroids are indicated and annotated with the corresponding simulation time (ns) at which the centroid structure occurs. Arrows denote transitions between dominant conformational states along the simulation trajectory. 15

Figure 13. Heatmap representation of hydrogen bond bond life (%) for the co-crystallized complex (4A5S) and all ligand-bound systems over the full simulation trajectories. Values indicate cumulative hydrogen bond occupancy for each residue–compound pair, highlighting persistent interactions and their relative contributions across systems...... 16

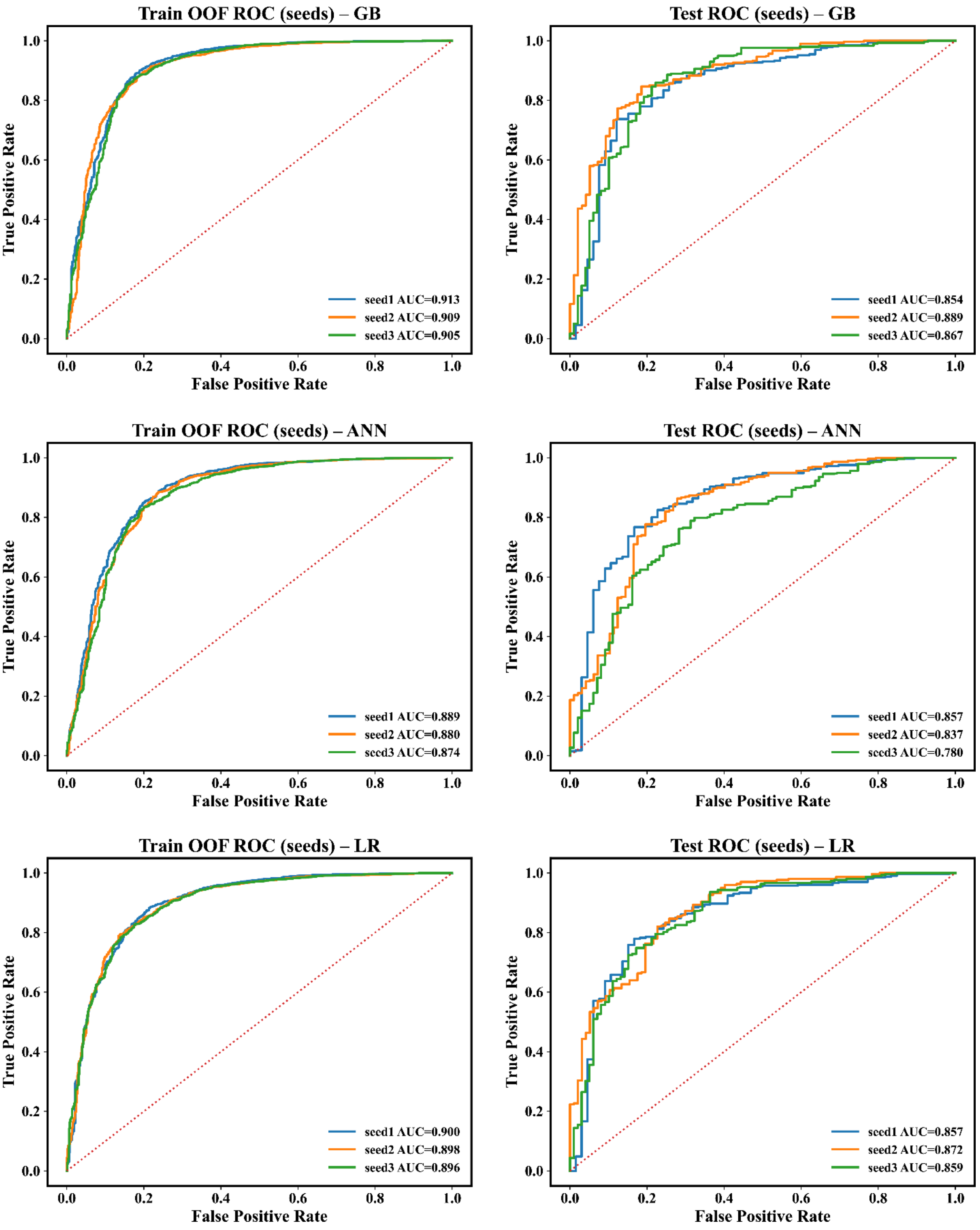

Figure S1. Receiver operating characteristic curves for logistic regression, random forest, gradient boosting, and artificial neural network models evaluated using three independent scaffold-based splits. For each model, curves are shown for out-of-fold training predictions and for held-out test sets.

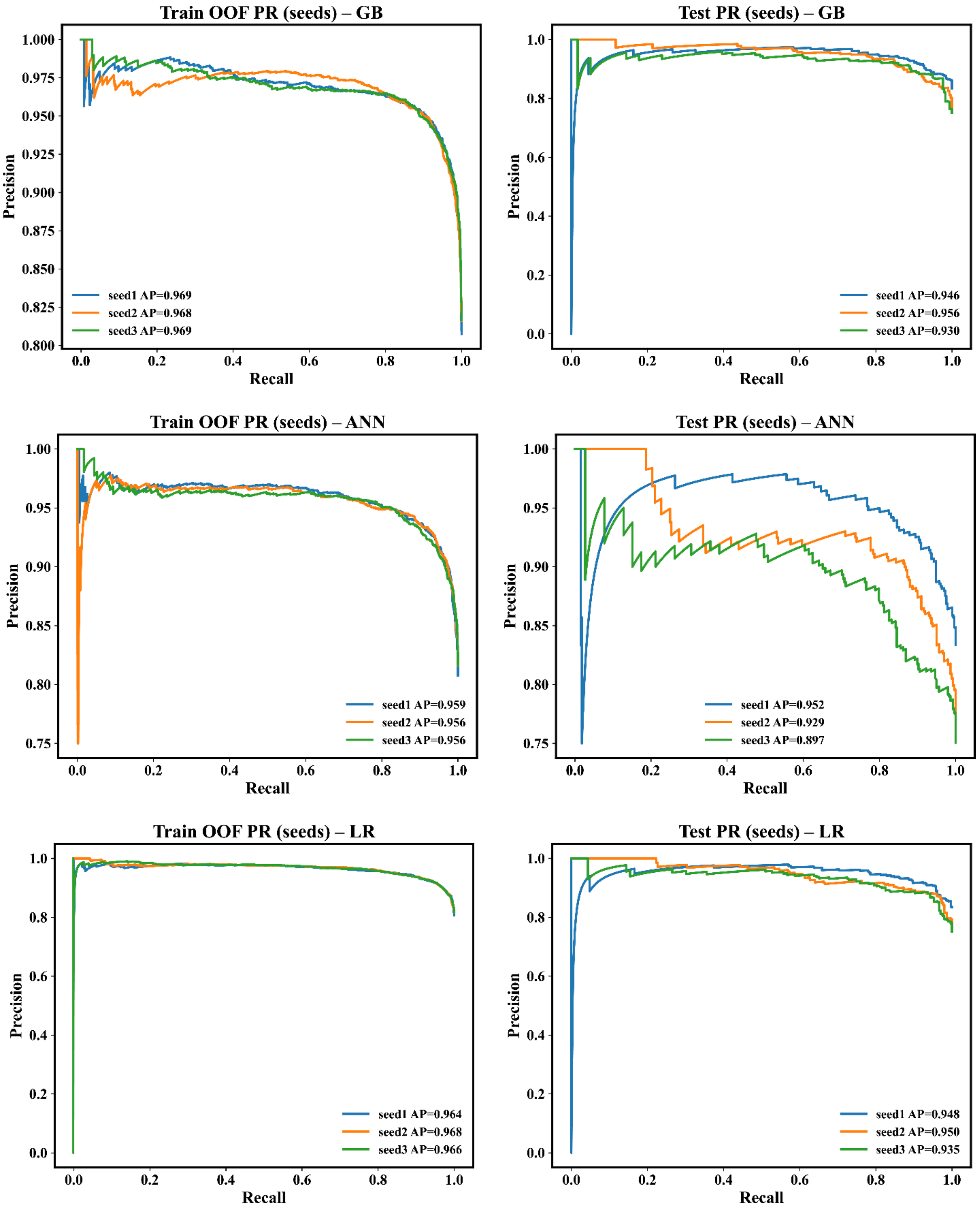

Figure S2. Precision–recall curves for logistic regression, random forest, gradient boosting, and artificial neural network models evaluated using three independent scaffold-based splits.

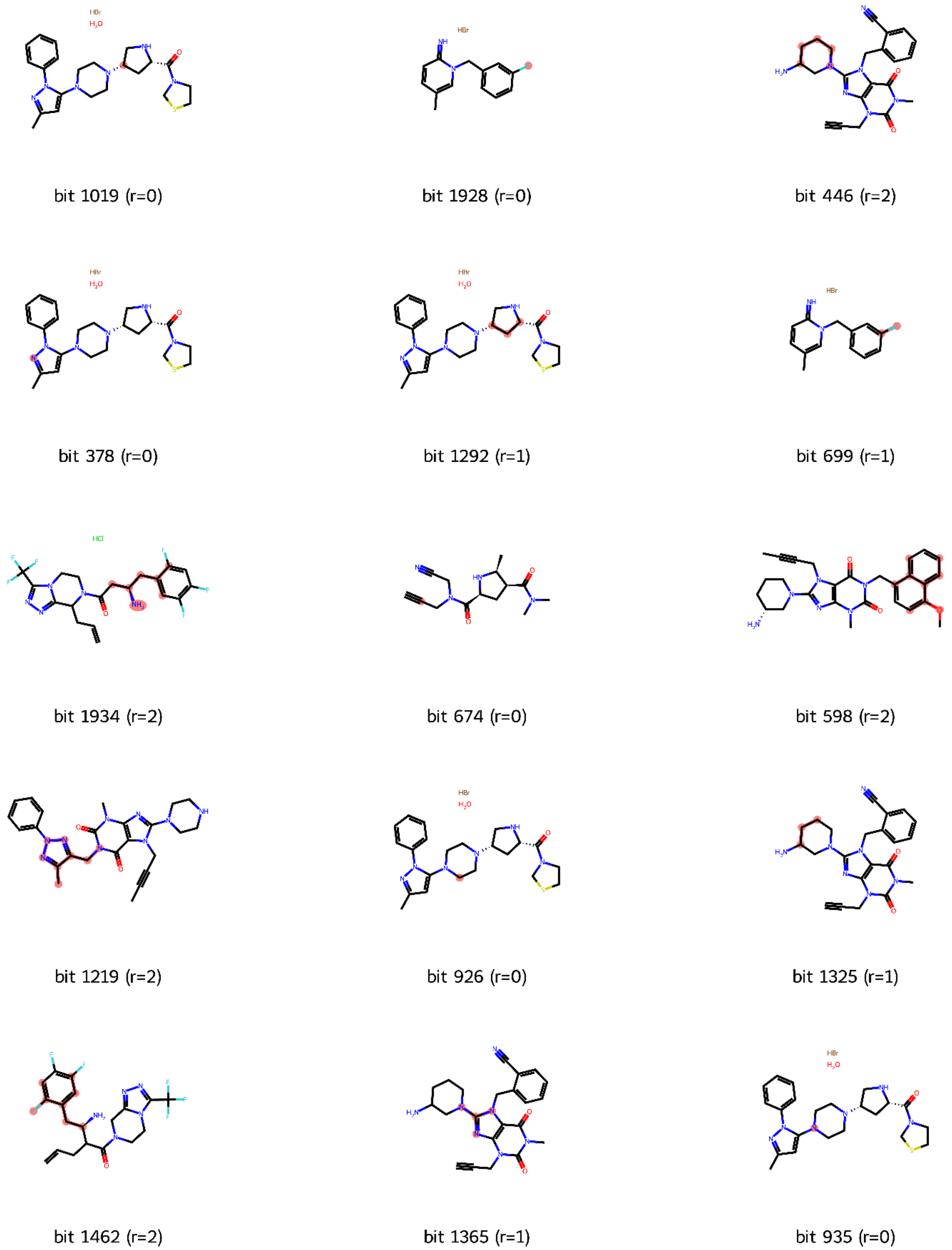

Figure S3A. Visualization of the thirty highest-ranking fingerprint substructures based on mean SHAP value. Each substructure is annotated by fingerprint bit index and radius, illustrating recurrent local chemical environments and shape features contributing to the random forest model output.

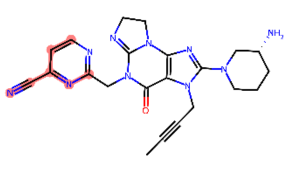

bit 1639 (r=2)

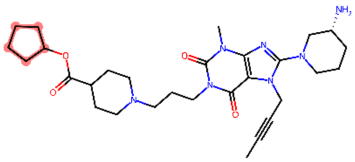

bit 890 (r=2)

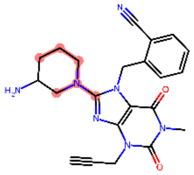

bit 680 (r=2)

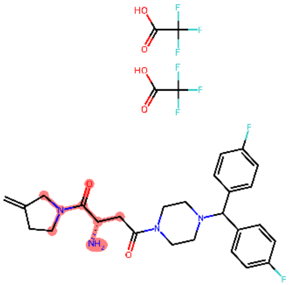

bit 196 (r=2)

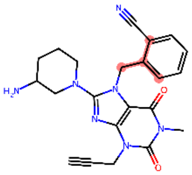

bit 1349 (r=1)

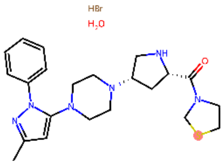

bit 675 (r=0)

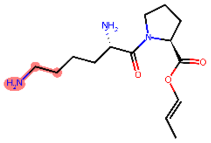

bit 1840 (r=1)

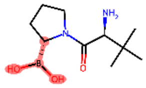

bit 911 (r=1)

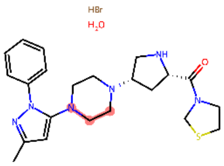

bit 1480 (r=1)

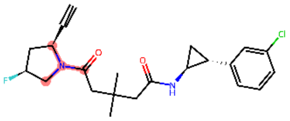

bit 242 (r=1)

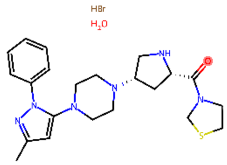

bit 650 (r=0)

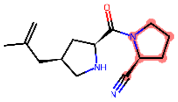

bit 454 (r=2)

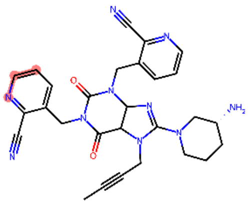

bit 1866 (r=1)

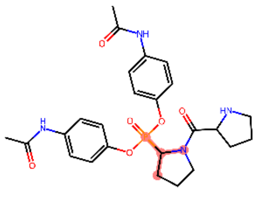

bit 1038 (r=1)

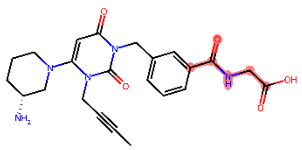

bit 1548 (r=2)

Figure S3B. Visualization of the thirty highest-ranking fingerprint substructures based on mean SHAP value. Each substructure is annotated by fingerprint bit index and radius, illustrating recurrent local chemical environments and shape features contributing to the random forest model output.

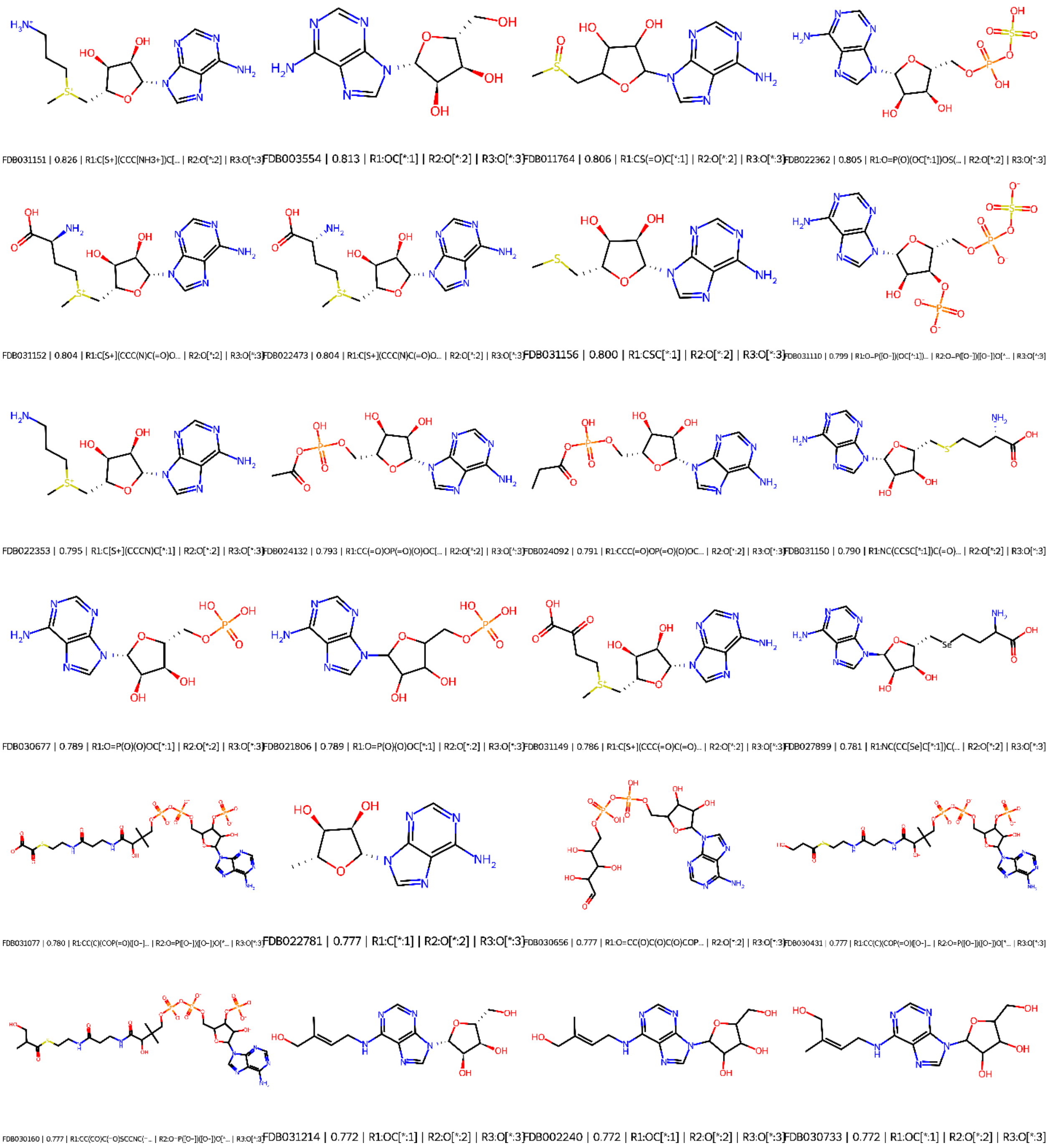

Figure S4. R-group analysis of representative high-scoring compounds identified by the random forest model, highlighting conserved core scaffolds and variable peripheral substituents. Predicted scores are reported for each compound, and substituent variation is shown relative to shared cores to illustrate structural diversity among top-ranked predictions.

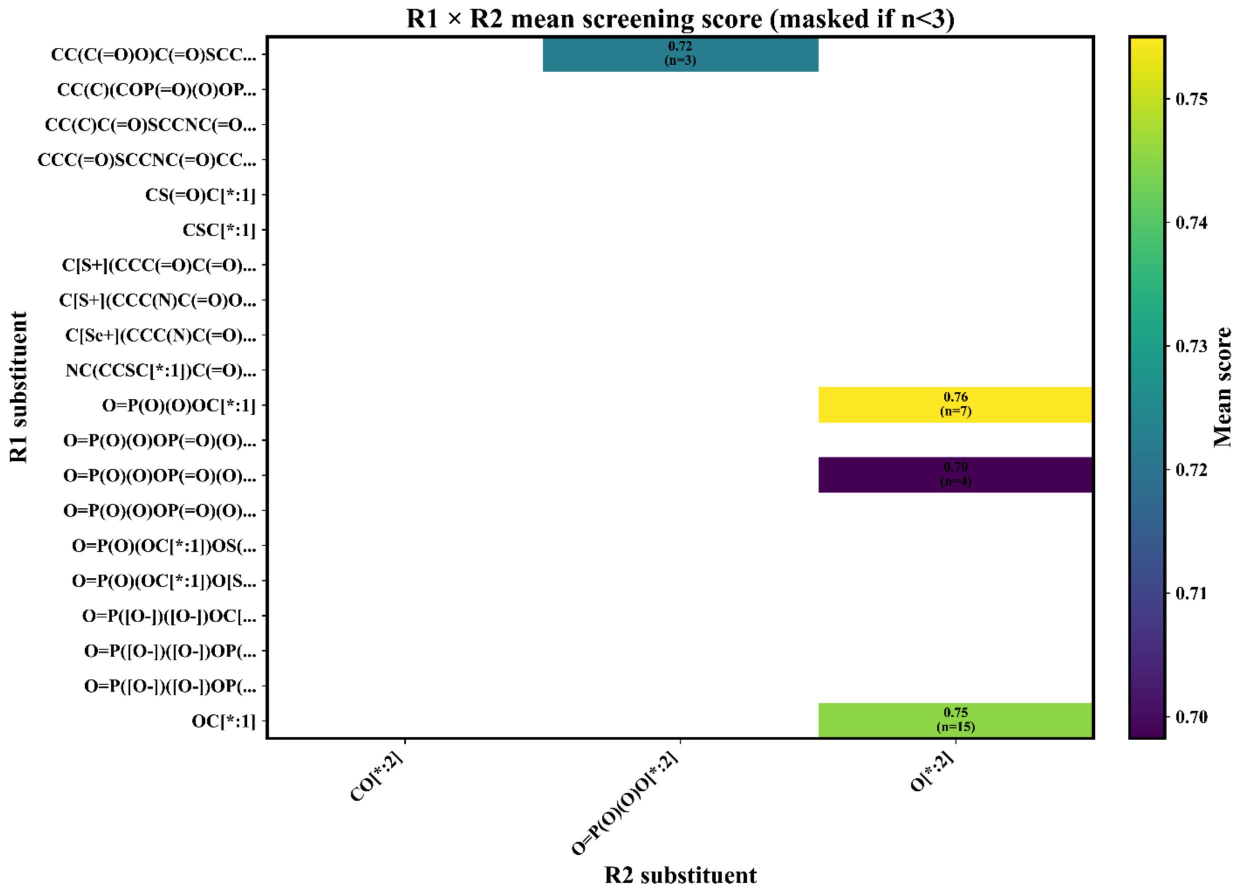

Figure S5. Heatmap showing mean screening scores for observed combinations of R1 and R2 substituents among high-scoring compounds predicted by the top-performing model. Only substituent pairs represented by at least three compounds are shown; combinations with fewer observations were masked. Mean scores are annotated within each cell together with the number of contributing compounds.

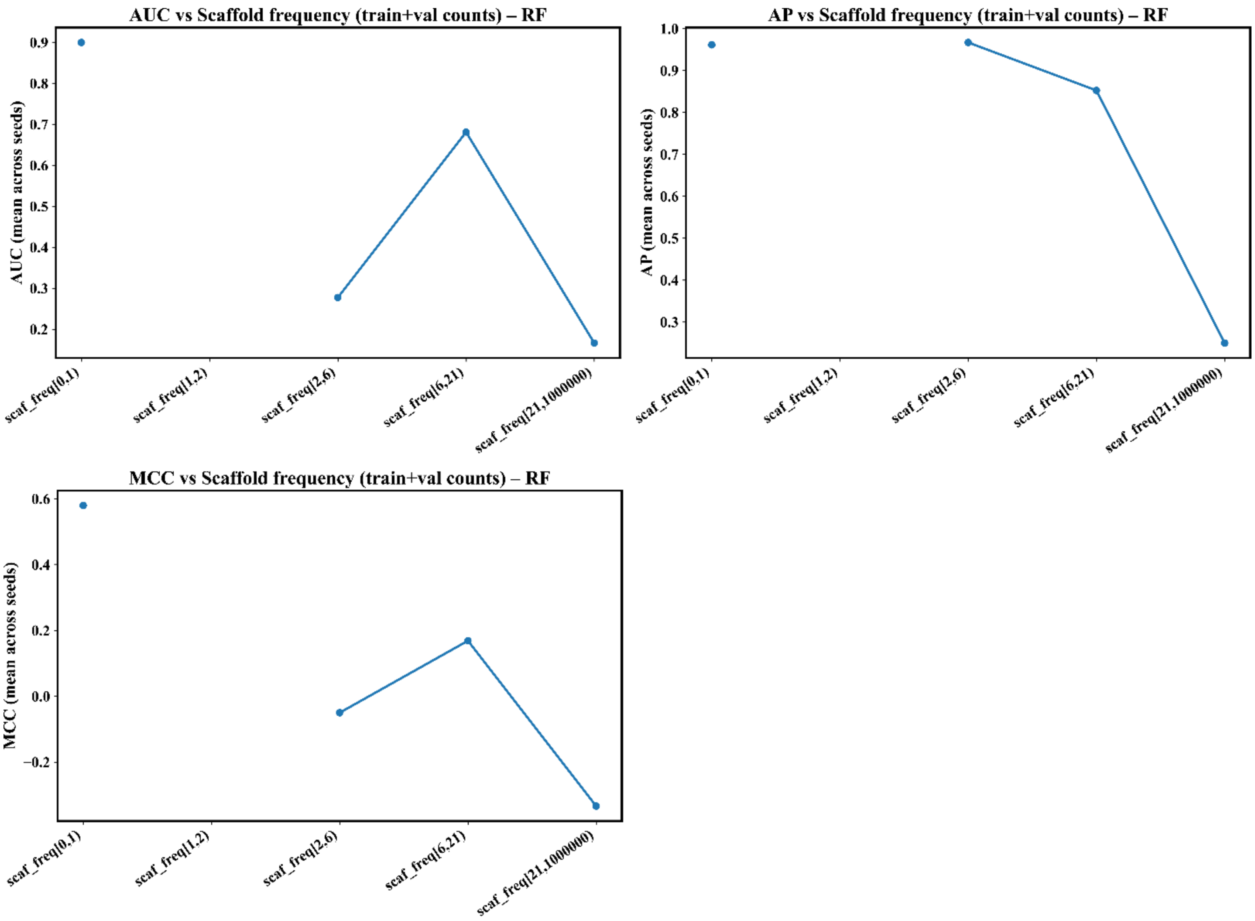

Figure S6. Mean test-set performance of the random forest classifier evaluated across scaffold frequency bins defined by the number of occurrences of each scaffold in the combined training and validation data. Panels report area under the receiver operating characteristic curve, average precision, and Matthews correlation coefficient, averaged across three independent scaffold split seeds.

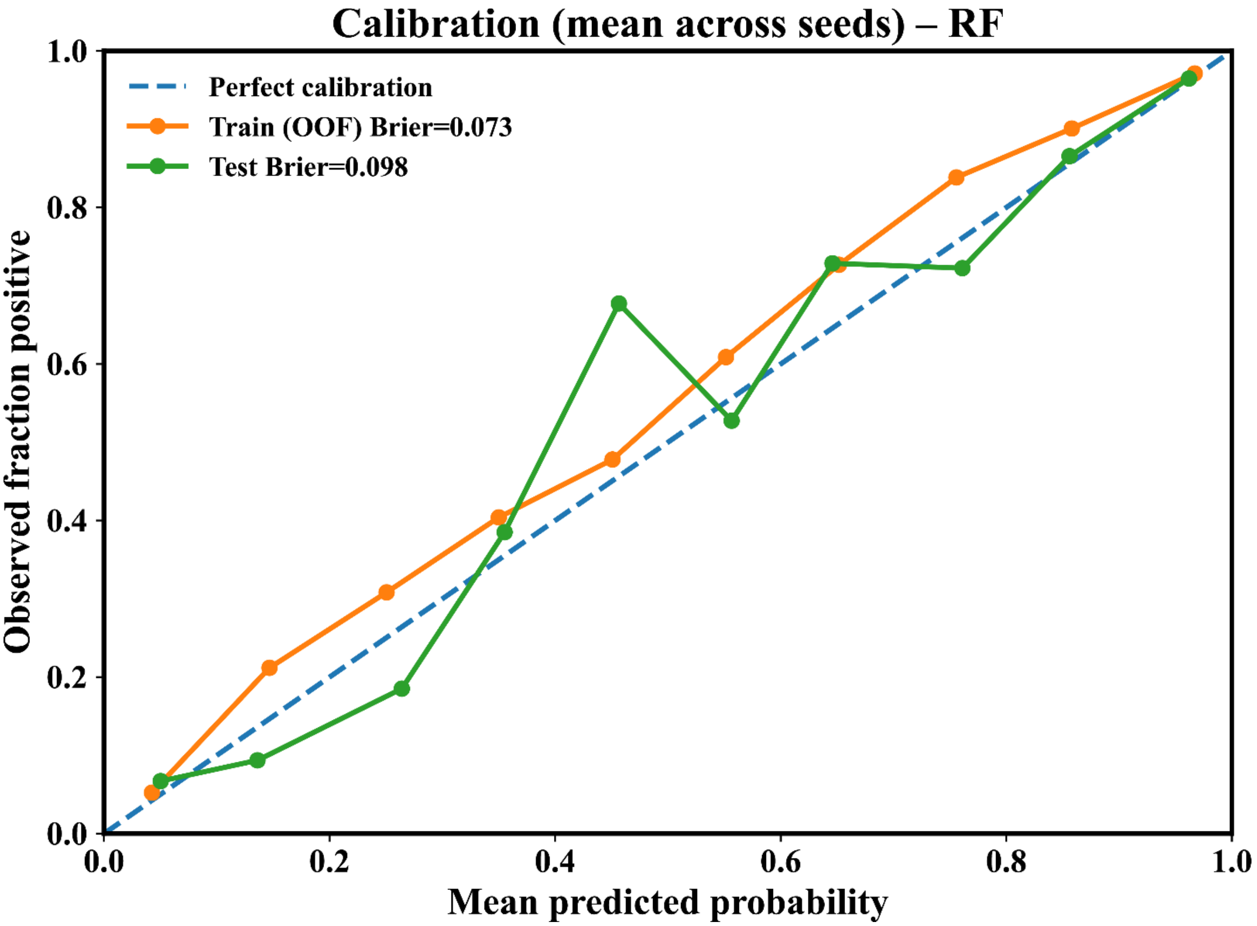

Figure S7. Calibration curves for the random forest classifier showing the relationship between mean predicted probability and observed positive fraction. Curves are shown for out-of-fold training predictions and for held-out test predictions, averaged across three scaffold split seeds. The dashed diagonal indicates perfect calibration, and corresponding Brier scores are reported in the legend.

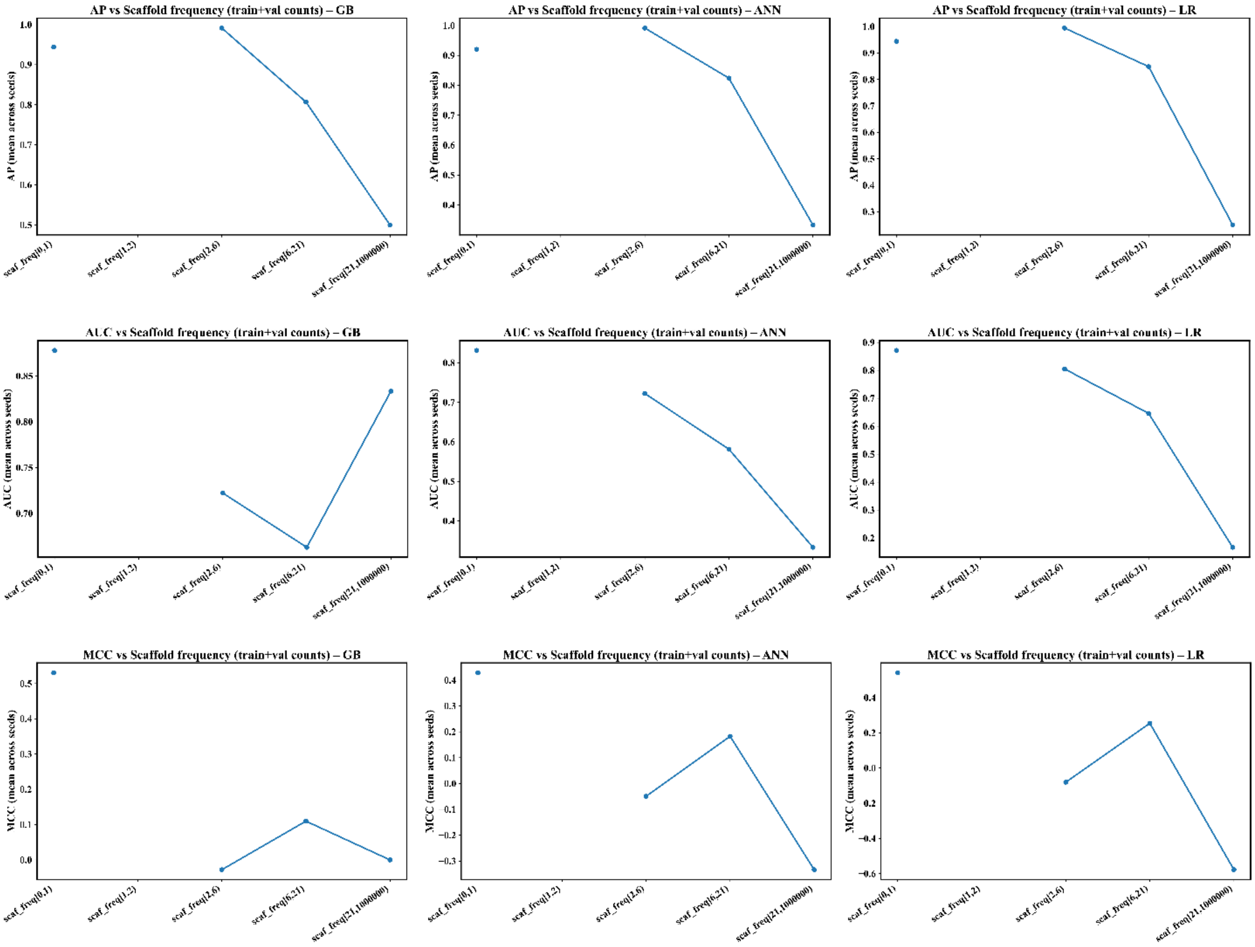

Figure S8. Mean test-set performance of gradient boosting, artificial neural network, and logistic regression classifiers evaluated across scaffold frequency bins defined by the number of occurrences of each scaffold in the combined training and validation data. Panels report average precision, area under the receiver operating characteristic curve, and Matthews correlation coefficient, averaged across three independent scaffold split seeds.

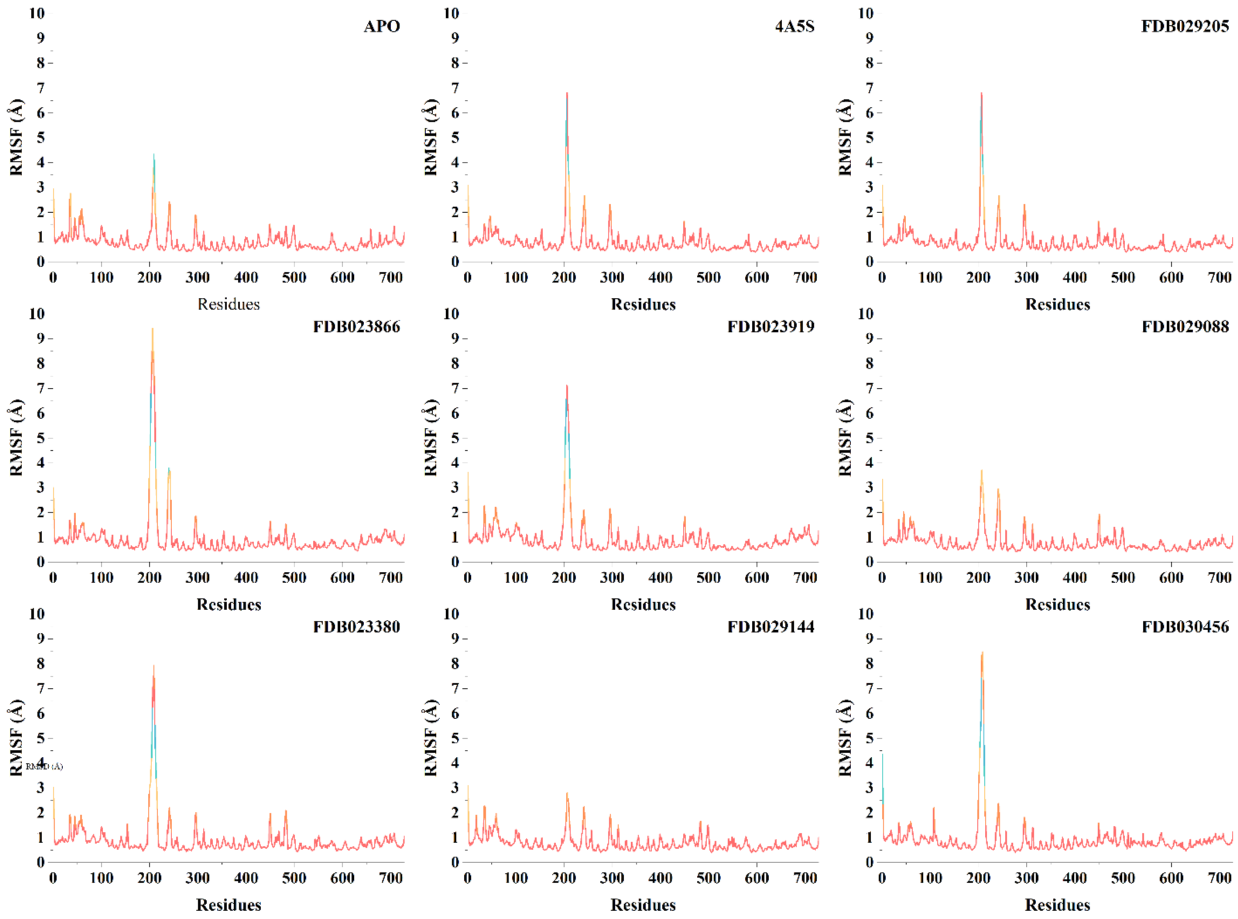

Figure S9. Root-mean-square fluctuation profiles of DPP4 residues for the apo protein, the co-crystallized complex (4A5S), and complexes with the seven selected food-derived compounds over 500 ns of simulation.

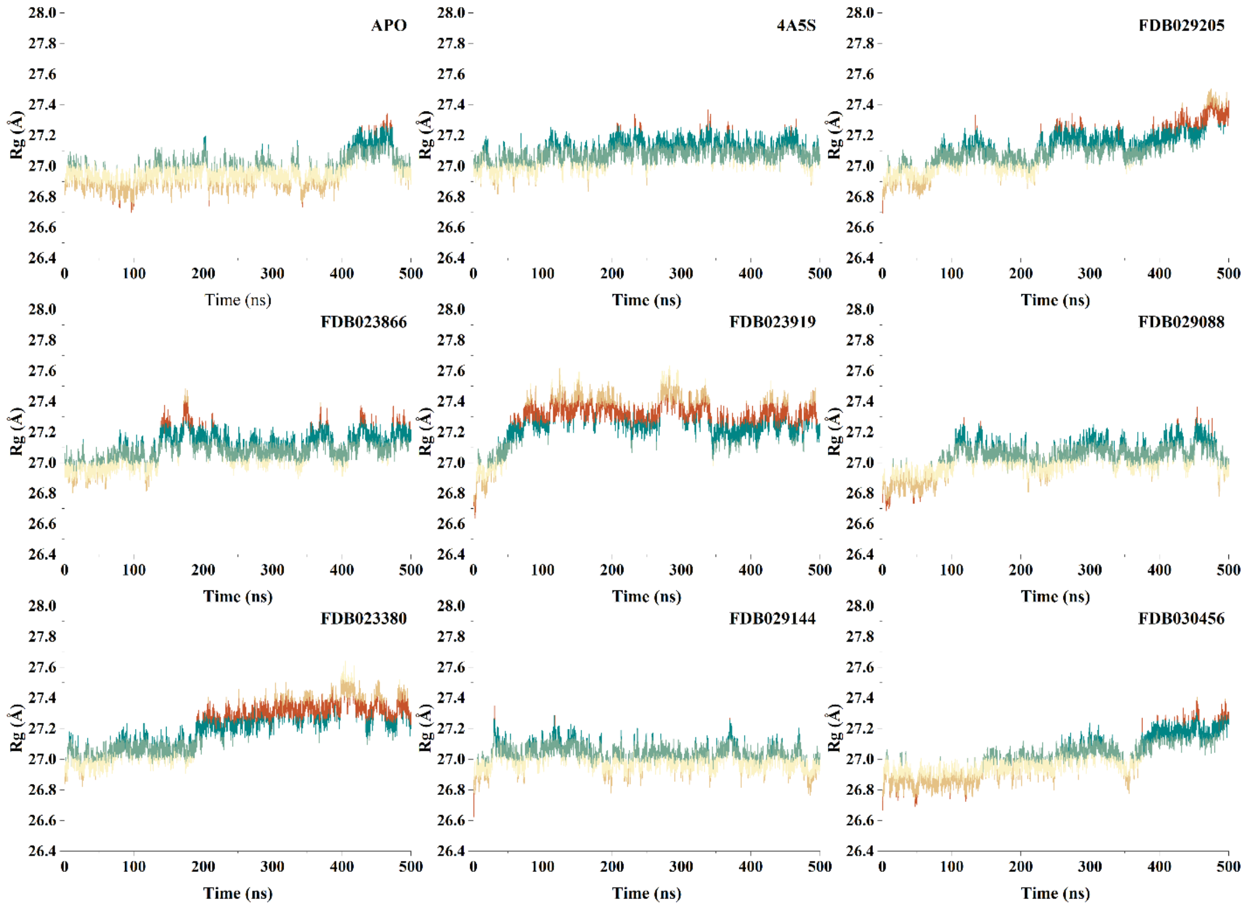

Figure 10. Radius of gyration (Rg) profiles of DPP4 over 500 ns molecular dynamics simulations for the apo protein, the co-crystallized complex (4A5S), and the seven ligand-bound systems.

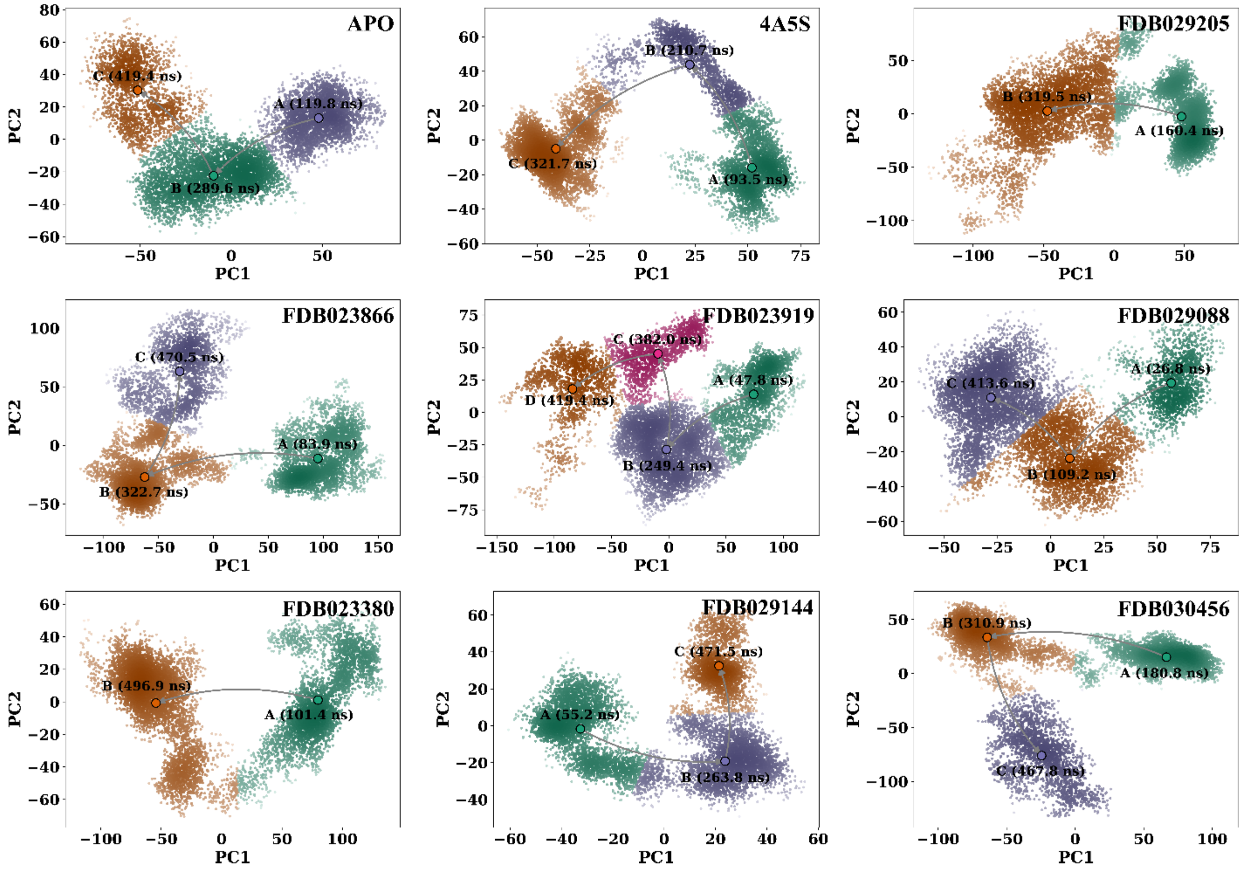

Figure 11. Two-dimensional projections of the molecular dynamics trajectories onto the first two principal components (PC1 and PC2) for the apo protein, the co-crystallized complex (4A5S), and the seven ligand-bound systems. Each point represents a trajectory frame colored according to k-means clustering. Cluster centroids are indicated and annotated with the corresponding simulation time (ns) at which the centroid structure occurs. Arrows denote transitions between dominant conformational states along the simulation trajectory.

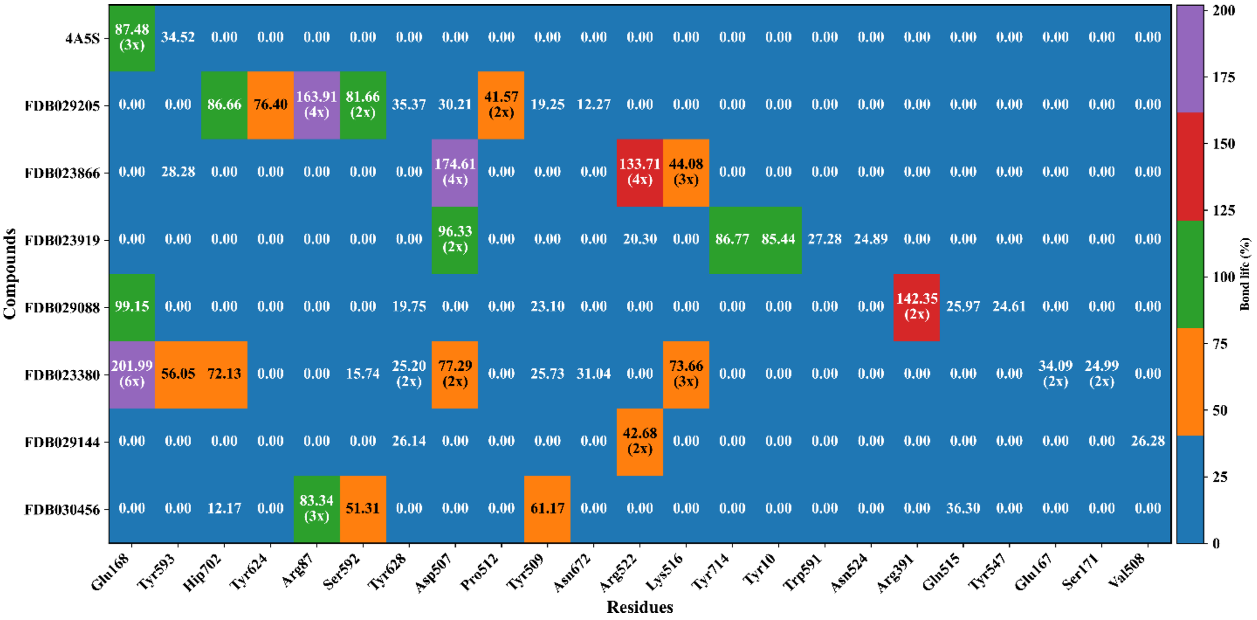

Figure 12. Heatmap representation of hydrogen bond bond life (%) for the co-crystallized complex (4A5S) and all ligand-bound systems over the full simulation trajectories. Values indicate cumulative hydrogen bond occupancy for each residue–compound pair, highlighting persistent interactions and their relative contributions across systems.
